# Two zinc finger proteins, VdZFP1 and VdZFP2, interact with VdCmr1 to promote melanized microsclerotia development and stress tolerance in *Verticillium dahliae*

**DOI:** 10.1186/s12915-023-01697-w

**Published:** 2023-10-31

**Authors:** Huan Li, Ruo-Cheng Sheng, Chen-Ning Zhang, Li-Chao Wang, Min Li, Ya-Hong Wang, Yu-Hang Qiao, Steven J. Klosterman, Jie-Yin Chen, Zhi-Qiang Kong, Krishna V. Subbarao, Feng-Mao Chen, Dan-Dan Zhang

**Affiliations:** 1 Co-Innovation Center for Sustainable Forestry in Southern China, Nanjing Forestry University, Nanjing, 210037 Jiangsu China; 2 State Key Laboratory for Biology of Plant Diseases and Insect Pests, Institute of Plant Protection, Chinese Academy of Agricultural Sciences, Beijing, 100193 China; 3 United States Department of Agriculture, Agricultural Research Service, Salinas, CA USA; 4 Western Agricultural Research Center, Chinese Academy of Agricultural Sciences, Changji, 831100 China; 5 Department of Plant Pathology, University of California, Davis, c/o United States Agricultural Research Station,, Salinas, CA USA

**Keywords:** *Verticillum dahliae*, Melanin, Zinc finger protein, Microsclerotia, Stress tolerance

## Abstract

**Background:**

Melanin plays important roles in morphological development, survival, host–pathogen interactions and in the virulence of phytopathogenic fungi. In *Verticillum dahliae*, increases in melanin are recognized as markers of maturation of microsclerotia which ensures the long-term survival and stress tolerance, while decreases in melanin are correlated with increased hyphal growth in the host. The conserved upstream components of the VdCmr1-regulated pathway controlling melanin production in *V. dahliae* have been extensively identified, but the direct activators of this pathway are still unclear.

**Results:**

We identified two genes encoding conserved C2H2-type zinc finger proteins VdZFP1 and VdZFP2 adjacent to *VdPKS9*, a gene encoding a negative regulator of both melanin biosynthesis and microsclerotia formation in *V. dahliae.* Both *VdZFP1* and *VdZFP2* were induced during microsclerotia development and were involved in melanin deposition. Their localization changed from cytoplasmic to nuclear in response to osmotic pressure. VdZFP1 and VdZFP2 act as modulators of microsclerotia melanization in *V. dahliae*, as confirmed by melanin biosynthesis inhibition and supplementation with the melanin pathway intermediate scytalone in albino strains. The results indicate that VdZFP1 and VdZFP2 participate in melanin biosynthesis by positively regulating *VdCmr1*. Based on the results obtained with yeast one- and two-hybrid (Y1H and Y2H) and bimolecular fluorescence complementation (BiFC) systems, we determined the melanin biosynthesis relies on the direct interactions among VdZFP1, VdZFP2 and VdCmr1, and these interactions occur on the cell walls of microsclerotia. Additionally, *VdZFP1* and/or *VdZFP2* mutants displayed increased sensitivity to stress factors rather than alterations in pathogenicity, reflecting the importance of melanin in stress tolerance of *V. dahliae*.

**Conclusions:**

Our results revealed that VdZFP1 and VdZFP2 positively regulate *VdCmr1* to promote melanin deposition during microsclerotia development, providing novel insight into the regulation of melanin biosynthesis in *V. dahliae*.

**Supplementary Information:**

The online version contains supplementary material available at 10.1186/s12915-023-01697-w.

## Background

Melanin is a negatively charged and hydrophobic high molecular weight pigment ubiquitously found in the biosphere [1, 2]. Generally, based on the chemical precursors in the biosynthesis, melanin can be divided into eumelanin, pheomelanin, neuromelanin, allomelanin, and pyomelanin [3]. However, 1,8-dihydroxynaphthalene melanin (DHN melanin), L-3,4-dihydroxyphenylalanine melanin (L-DOPA melanin), and pyomelanin formed by various endogenous substrates are the ubiquitous types in the microbial kingdom [4–7]. The biosynthesis of melanin involves the enzymatic catalysis of phenols, quinones, or indoles, which are complexed with saccharides or proteins. These polymers are formed often in response to stimulation signals from the environment, such as cytotoxic components and/or homeostasis disorders, and thus are adaptations that acquire functions in stress tolerance, maintenance of dormancy, host infections, and pathogen-host interactions and in niche competition [2, 4, 8].

In ascomycete fungi, genes encoding polyketide synthases (PKS) are often co-located in clusters, with those genes encoding dehydratases and reductases that comprise the backbone enzymes of the DHN-melanin biosynthesis pathway [9]. Together these enzymes catalyze the conversion of acetyl-CoA to 1,3,6,8-tetrahydroxynaphthalene (1,3,6,8-THN), scytalone, 1,3,8-trihydroxynaphthalene (1,3,8-THN), vermelone, and finally 1,8-DHN [9, 10]. The intermediate products and the necessary enzymes are encapsulated and continuously externalized at the fungal cell wall where the 1,8-DHN is finally catalyzed into melanin macromolecules and deposited [11]. DHN-melanin contributes to the ability of melanin-producting fungi to survive in harsh environments. The black yeasts *Hortaea werneckii*, *Trimmatostroma salinum*, and *Phaeotheca triangularis* rely on DHN-melanin to adapt to hypersaline environment and maintain cell wall integrity and normal cell division [4, 12].

For pathogenic fungi, the conserved DHN-melanin pathway is a prerequisite for the development of invasive structures, resistant conidia, and mature (micro)sclerotia. For example, albino mutants lose their pathogenicity due to the lack of melanin in appressoria of *Magnaporthe oryzae* and *Colletotrichum gloeosporioides* [13, 14]. Melanin-deposited in the appressoria ensures the accumulation of osmotic substances and high turgor in appressoria to produce functional penetration pegs [13, 14]. Aside from its direct role in pathogenicity in some fungi, melanin mediates growth and stress tolerance of conidia and sclerotia in *Botrytis cinerea* [15, 16]. Moreover, melanin in conidia of *Aspergillus fumigatus* interferes with host endocytosis pathways recognized by the human MelLec receptor, activating hypoxia-inducible factor 1 subunit alpha and recruiting phagosomes to drive antifungal immunity [17]. During this process, melanin can block host cytotoxicity by effectively inhibiting lysosome acidification [18, 19].

Melanin biosynthesis in pathogenic fungi is regulated by transcription factors (TFs) with different functional mechanisms. Regulation of melanin biosynthesis by the calcineurin-responsive C2H2-type zinc finger TF Crz1p and its homologs is well-studied [20–22]. The Cmr1p-like protein is another conserved TF that regulates melanin biosynthesis gene clusters in *M. oryzae*, *B. cinerea*, *Cochliobolus heterostrophus*, *Setosphaeria turcica*, and *Alternaria brassicicola* [23–27]. Besides these TFs, many specialized TFs mediate expression of the melanin biosynthesis pathway. For example, DevR and RlmA of *A. fumigatus* can recognize the DNA motifs of *pksP* promoter region and regulate it by acting as repressors and activators [28]. In *Pestalotiopsis fici*, PfmaF stimulates PfmaH to positively regulate the expression of scytalone dehydratase [29]. The contribution of melanin-related TFs to pathogenicity likely depends upon their regulatory coverage and in how their coverage affects melanin localization. The silencing of SsFkh1 leads to the downregulation of melanin-related genes and the unavailability of sclerotia that are essential for *S. sclerotiorum* to invade the host and long-term survival [30]. For *M. oryzae*, MoSwi6 interacts with MoMps1 to mediate melanization of appressorium, conidiation, cell wall integrity, and virulence, while over-melanization of a MoZFC3 deletion mutant regulated MAP1-mediated pathogenicity [31, 32]. However, BcZTFs are responsible for the accumulation of melanin in conidia of *B. cinerea* rather than participating in pathogenesis [10].

*Verticillium dahliae* is a notorious hemi-biotrophic pathogen causing Verticillium wilt of more than 200 dicotyledonous plants including economically important agricultural crops and trees [33–35]. The coevolution between pathogens and host plants has prompted the kaleidoscopic differentiation of the population structure of *V. dahliae* into defoliating and nondefoliating phenotypes, physiological races, clonal populations, and populations with different mating type genes [36, 37]. The development of melanized microsclerotia is recognized as a unique morphological characteristic among *Verticillium* fungi, with *V. dahliae* naturally being differentiated into hyphal- and microsclerotia-type strains. Melanized microsclerotia form both in experimental conditions and the necrotic host tissues [34, 38, 39]. The formation of microsclerotia in *V. dahliae* is initiated from swollen hyphae, and these initial structures become condensed with thickened walls and are heavily pigmented [40]. *V. dahliae* is notoriously difficult to combat owing to heavily melanized microsclerotia, which resist UV radiation and high temperature, maintaining vitality for 14 years and serve as initial inoculum for the disease cycle [9, 38, 41]. In response to host root secretions, the microsclerotia germinate and form hyphae to invade roots, breach the cortex, and proliferate in the xylem [37, 42]. Once infected, *V. dahliae* causes wilting, vascular tissue discoloration and even death [43, 44]. Thus, clarifying the regulatory bases of the development of melanized microsclerotia is of vital importance for targeted control strategies of *V. dahliae*.

Studies that have identified and functionally characterized *V. dahliae* TFs and their associated signaling pathways have focused on their relationships with microsclerotia and pathogenicity. Mitogen-activated protein kinase (MAPK) cascades are conserved signal transduction components that regulate specific genes with multiple biological functions in eukaryotes, including stress adaptation, proliferation, and differentiation [45, 46]. In MAP kinase pathways elucidated in *V. dahliae*, the downstream C2H2-type zinc finger protein VdMsn2 [47] and the MADS-Box TF VdMcm1 [48] contribute to growth, melanin biosynthesis, microsclerotia formation, and pathogenicity. However, though not its only role, *VdHog1*-governed VdCmr1 is responsible for the regulation of the *VdPKS1* gene cluster to biosynthesize melanin [9]. The fungal-specific Zn(II)_2_Cys_6_-type TF Vdpf is a mediator of G protein-mediated and cAMP-dependent protein kinase A (PKA) pathways, both of which affect melanin production, microsclerotia formation, and virulence in *V. dahliae* [49–51]. Moreover, signaling pathway regulators VdAtf1, VdYap1, and VdSkn7 are involved in reactive oxygen/nitrogen species (ROS/RNS) response, nitrogen utilization, microsclerotia formation, and virulence [52]. The bZip TF VdMRTF1 [53] and transcription co-activator complex subunit VdAda1 [54] were confirmed to play important roles in melanin biosynthesis, microsclerotia development, and pathogenicity. Therefore, the relationships among microsclerotia formation, virulence, and melanin biosynthesis are complicated and these physiological processes are modulated by pathway cross-talk [38]. However, the regulatory elements that directly mediate the maturation of the microsclerotia in *V. dahliae* are still obscure.

In a previous study, we identified VdPKS9 which regulates morphological differentiation in *V. dahliae* and acts as a negative regulator of microsclerotia and melanin biosynthesis in *V. dahliae* while its overexpression promotes non-melanized hyphal growth [39]. Herein, two C2H2-type *z*inc *f*inger *p*roteins (VdZFP1 and VdZFP2) located upstream of *VdPKS9* were demonstrated to regulate melanin biosynthesis in *V. dahliae*. Further, VdZFP1 and VdZFP2 were localized in the nucleus following exposure to osmotic stress and positively regulated *VdCmr1* to mediate melanin deposition in microsclerotia. Intriguingly, VdZFP1 and VdZFP2 were shown to physically interact with VdCmr1 on the cell wall, suggestive of a novel regulatory mechanism of melanin production. VdZFP1 and VdZFP2 contributed to the stress tolerance but not to the pathogenicity of *V. dahliae*. Taken together, our findings reveal important regulatory roles for VdZFP1 and VdZFP2 in the development of melanized microsclerotia in *V. dahliae*, which broadens our understanding of the mechanisms of fungal melanin regulation.

## Results

### Two C2H2-type zinc finger proteins upstream of the putative VdPKS9 gene cluster are conserved in filamentous fungi

**Fig. 1:**
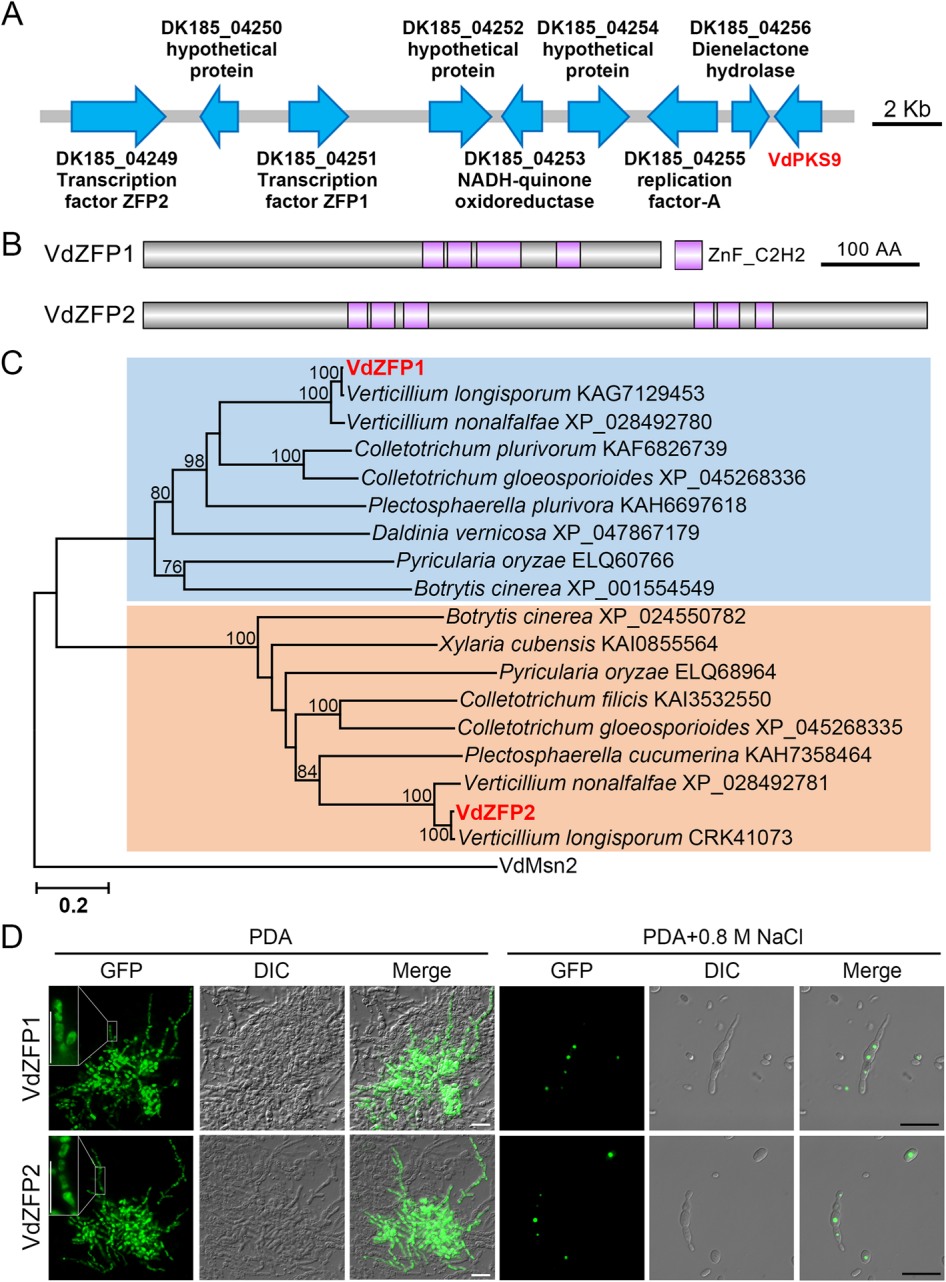
VdZFP1 and VdZFP2 with variable subcellular localization upstream of *Verticillium dahliae* VdPKS9 are conserved in filamentous fungi. **A** The putative *VdPKS9* gene cluster in *V. dahliae*. A sketch map of gene cluster covering eight upstream genes of *VdPKS9* was constructed based on genome sequencing and annotation results of *V. dahliae* AT13 strain. Each arrow represents the direction of gene expression. Scale bar = 2000 bp. **B** The C2H2-type zinc finger motifs of VdZFP1 and VdZFP2. The protein sequences were predicted by multiple pipelines of SMART, InterPro, and Pfam and zinc finger motifs were labeled in purple. Scale bar = 100 amino acids. **C** Phylogenetic analysis of VdZFP1 and VdZFP2 in *V. dahliae* and their homologs from other melanin-producing fungi. The protein sequences were downloaded from NCBI database. MEGA 7.0 software was used to construct the phylogenetic tree based on the neighbor-joining method. The reliabilities indicated at the branch nodes were evaluated using 1000 bootstrap replications. **D** Subcellular localization of VdZFP1 and VdZFP2 in *V. dahliae*. The VdZFP1- and VdZFP2-GFP fragments were introduced into the genome of WT strain, respectively and the transformants were cultured on PDA medium (or supplemented with 0.8 M NaCl) for 4 days. The GFP signals were observed by fluorescence microscopy. Scale bar = 20 μm

PKS gene clusters usually contain TFs to specifically regulate the biosynthesis of secondary products, such as the fumonisin and fusaric acid metabolic clusters [55, 56]. Therefore, a 22,235-bp fragment including 9 genes was defined as a putative *VdPKS9* gene cluster for investigation, which encodes two ZFPs (DK185_04249 and DK185_04251 in strain AT13 (https://db.cngb.org/Verticilli-Omics/); VDAG_08644 and VDAG_08646 in strain VdLs.17 [34]), one NADH-ubiquinone oxidoreductase (DK185_04253), one replication factor (DK185_04255), one diene-lactone hydrolase (DK185_04256), VdPKS9, and three other hypothetical proteins (DK185_04250, DK185_04252, DK185_04254) (Fig. 1A). ZFPs were given priority because of their key functions in fungal physiological and biochemical processes. According to the gene loci, two *ZFPs* were named VdZFP1 (DK185_04251) and VdZFP2 (DK185_04249). VdZFP1 and VdZFP2 encode 585 and 886 aa (amino acids), respectively, and were predicted to possess four and six C2H2-type zinc finger domains, respectively, based on the SMART and InterPro online tools (Fig. 1B). Furthermore, phylogenetic analysis showed that VdZFP1 and VdZFP2 shared highest sequence identities with *Verticillium* spp. homologs while phylogenetic analysis revealed that VdZFP1 and VdZFP2 were conserved in other melanin-producing filamentous fungi (Fig. 1C).

To determine the subcellular localization of the two ZFPs, VdZFP1 and VdZFP2 were fused with the green fluorescent protein (GFP) and transferred into the highly-virulent strain AT13 (wild-type (WT) strain). Surprisingly, both were present in the cytoplasm of swollen hyphae and germinating conidia of *V. dahliae* (Fig. 1D). Since environmental conditions may affect nuclear localization of VdZFP1 and VdZFP2, the localization of both was also examined in response to osmotic stress. Following incubation on PDA medium supplemented with 0.8 M NaCl, GFP signals of VdZFP1 and VdZFP2 were observed in the nucleus of both hyphae and conidia (Fig. 1D). Thus, the variable subcellular localization of VdZFP1 and VdZFP2 in *V. dahliae* suggested that VdZFP1 and VdZFP2 may function in specific stress responses and potentially microsclerotia development in *V. dahliae*.

### VdZFP1 and VdZFP2 are involved in melanin production

**Fig. 2:**
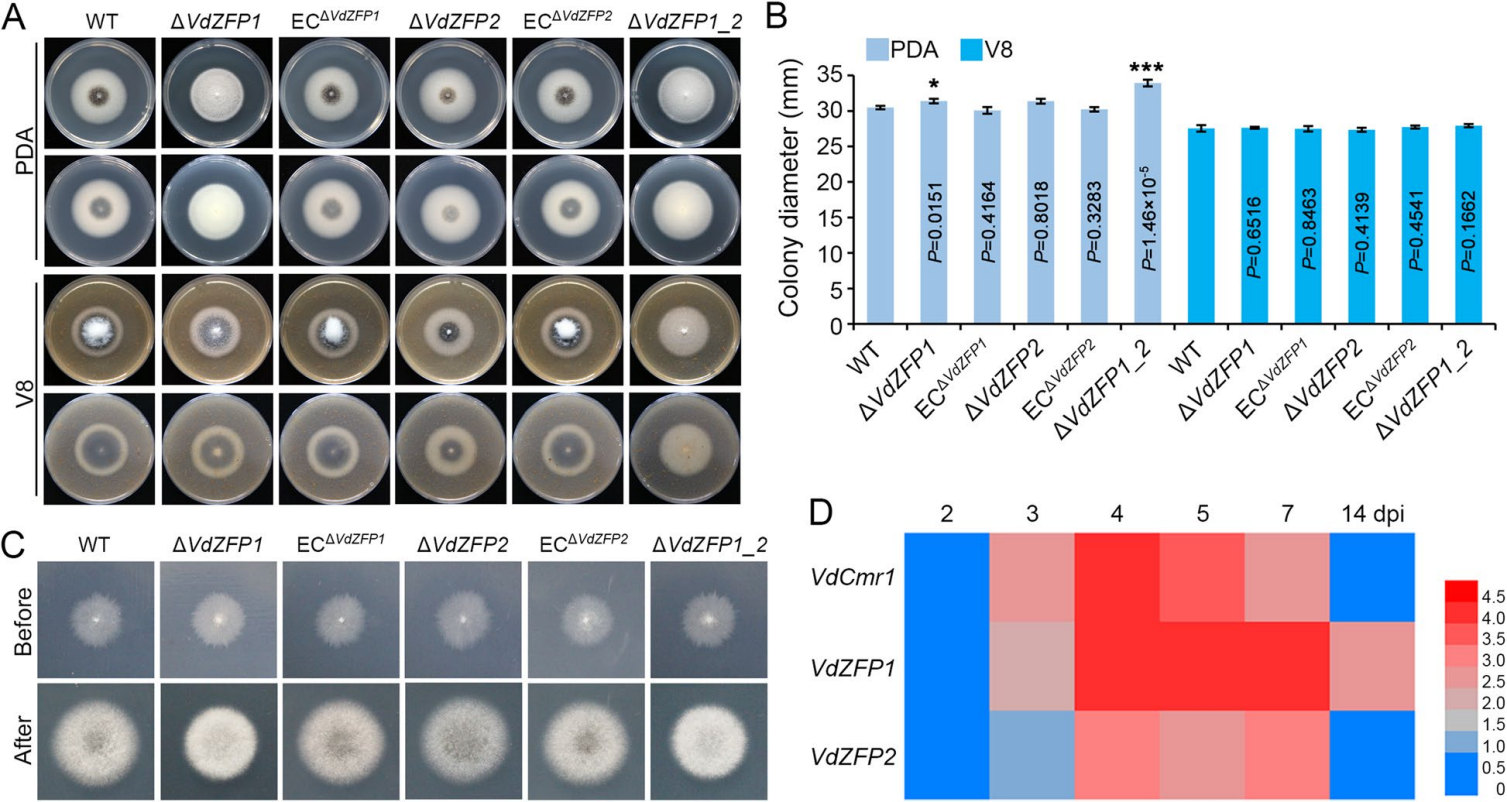
VdZFP1 and VdZFP2 play key roles in melanin production of *Verticillium dahliae*. **A** Colony morphology of WT, Δ*VdZFP1*, EC^Δ*VdZFP1*^, Δ*VdZFP2*, EC^Δ*VdZFP2*^, and Δ*VdZFP1_2* inoculated on PDA and V8 media at 25 °C in the dark. The phenotypes were photographed 7 days after incubation. Each strain was inoculated at least three plates and three independent experiments were carried out. **B** Colony diameters of the different strains in panel **A**. Error bars are standard errors calculated from six replicates, and this experiment performed three repeats. ^*^P < 0.05, ^**^P < 0.01 (Student’s *t* test). **C** The penetrated ability of WT, Δ*VdZFP1*, EC^Δ*VdZFP1*^, Δ*VdZFP2*, EC^Δ*VdZFP1*^, and Δ*VdZFP1_2* on cellophane membranes. The hyphal blocks were cut from PDA plates and placed on MM medium covered with cellophane membranes at 25 °C in the dark. The cellophane membranes were removed at 3 dpi, and the treated plates were incubated for an additional 5 days. This experiment performed three repeats. **D** Expression profile of *VdZFP1*, *VdZFP2*, and *VdCmr1* during microsclerotia development of *V. dahliae*. The conidia of WT strain were grown on BMM medium covered with cellophane membranes at 25 °C in the dark and samples were collected at 2, 3, 4, 5, 7, and 14 dpi. The relative expression of *VdZFP1*, *VdZFP2* and *VdCmr1* were calculated from the RT-qPCR results using the 2.^−ΔΔCT^ method with 2 dpi as control. This experiment was independently repeated 3 times to determine the trend. The results are presented in a heatmap (the expression levels were normalized to Log_2_ (fold-change))

To determine the importance of VdZFP1 and VdZFP2 on several phenotypes in *V. dahliae*, single- and double-deletion mutants (Δ*VdZFP1*, Δ*VdZFP2*, and Δ*VdZFP1_2*) were obtained in the WT strain by homologous recombination (Additional file 1: Figure S1A) and ectopic complemented transformants (EC^Δ*VdZFP1*^ and EC^Δ*VdZFP2*^) were also generated by reintroduction of *VdZFP1* and *VdZFP2* into the deletion mutants. All the mutants were verified by multiple diagnostic PCR assays (Additional file 1: Figure S1B – 1F). Subsequently, WT, deletion mutant, and complemented strains were inoculated onto PDA and V8 plates for analyses of growth and melanin production. Compared with the WT, *VdZFP1*-deleted strain produced albino colonies on PDA medium at 7 dpi but maintained melanin deposition on V8 medium (Fig. 2A). For the *VdZFP2*-deletion strain, the melanized area was reduced on both PDA and V8 medium compared to the WT (Fig. 2A). Complementation restored melanin production (Fig. 2A). The melanin phenotypes of the double-deletion strains showed extreme albinism on both media (Fig. 2A). These results indicated that VdZFP1 and VdZFP2 regulate melanin biosynthesis in *V. dahliae*. To eliminate the redundant regulatory functions of *VdZFP1* and *VdZFP2* in melanin biosynthesis, their expression levels were examined on PDA medium. Results from RT-qPCR suggested that *VdZFP1* and *VdZFP2* do not regulate the expression of one another during melanin biosynthesis (Additional file 1: Figure S2A). Moreover, the variable colony diameters of the individual deletion mutant *VdZFP1* or the *VdZFP1_2* double-deletion indicated that *VdZFP1* also participates in the growth and nutrient utilization of *V. dahliae* (Fig. 2B; Additional file 1: Figure S3).

Previous studies have shown that reductions in melanin biosynthesis in *V. dahliae* are sometimes correlated with reductions in hyphal penetration, and invasion, and impaired microsclerotia development [9, 57, 58]. To evaluate a similar correlation in the *VdZFP1* and *VdZFP2* mutant strains, the host epidermis penetration ability of WT, deletion mutants, and the complemented strains were examined on cellophane membranes overlaid on MM medium. The reduction in melanin of *VdZFP1*- and *VdZFP2*-deleted mutants was independent of penetration since removal of the cellophane membranes at 72 hpi (hours post inoculation) did not affect hyphal penetration and growth (Fig. 2C). Since microsclerotial maturation involves the deposition of melanin in WT *V. dahliae* [40], we associated the deficiency of melanin production to the lack of melanization of microsclerotia. To examine this, the expression patterns of *VdZFP1* and *VdZFP2* during microsclerotia formation of the WT strain on BMM medium were analyzed. Like *VdCmr1*, the expression levels of *VdZFP1* and *VdZFP2* were induced in various developmental phases of microsclerotia. Compared with *VdZFP2* and *VdCmr1*, *VdZFP1* showed higher transcript levels (Fig. 2D). Conversely, *VdZFP1* and *VdZFP2* were significantly inhibited in hyphae-type strain (Vd991) (Fig S2B). These results suggested that *VdZFP1* and *VdZFP2* are important for microsclerotia formation, and clarified that VdZFP1 and VdZFP2 are involved in melanin production during the formation of microsclerotia in *V. dahliae*.

### VdPKS9 functions independently of VdZFP1 and VdZFP2

**Fig. 3:**
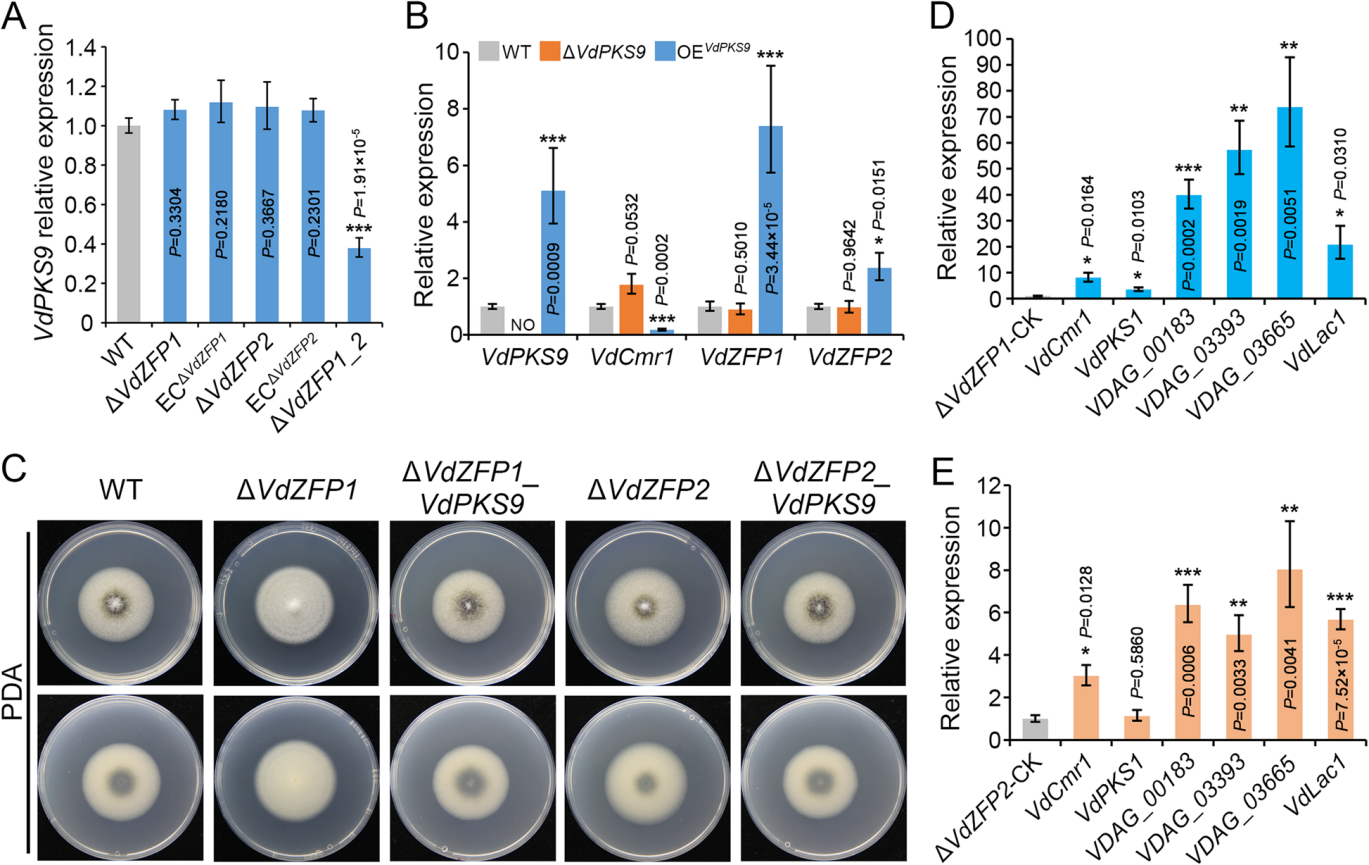
*Verticillium dahliae VdPKS9* is not governed by VdZFP1 and VdZFP2. **A** Relative expression of *VdPKS9* in WT, Δ*VdZFP1*, EC^Δ*VdZFP1*^, Δ*VdZFP2*, EC^Δ*VdZFP1*^, and Δ*VdZFP1_2* strains. These mutants were collected as RNA samples after induction on BMM medium at 25 °C in the dark for 5 days. **B** Relative expression of *VdCmr1*, *VdZFP1*, and *VdZFP2* in Δ*VdPKS9* and OE^*VdPKS9*^ strains. The RNA samples were collected on BMM medium 5 days after incubating at 25 °C in the dark. **C** Colony morphology of WT, Δ*VdZFP1*, Δ*VdZFP1_VdPKS9*, Δ*VdZFP2*, and Δ*VdZFP2_VdPKS9* inoculated on PDA medium. The phenotypes were photographed 7 days after incubation at 25 °C in the dark. Each strain was plated on at least three plates and three independent experiments were carried out. **D, E** Analyses of the relative expression of melanin biosynthesis genes in Δ*VdZFP1_VdPKS9* and Δ*VdZFP2_VdPKS9* by RT-qPCR. RNA samples were collected from the indicated strains that grown on BMM medium for 5 days, and the transcript level in two single-gene-deleted mutants (Δ*VdZFP1* and Δ*VdZFP2*) was used as control. Above experiments related to RT-qPCR detection were independently repeated 3 times, and the results were conducted using the 2^−ΔΔCT^ method. Error bars represent standard errors of the mean, and ^*^P < 0.05, ^**^P < 0.01, and.^***^P < 0.001 (one-way ANOVA)

Given that *VdZFP1*, *VdZFP2*, and *VdPKS9* belong to a putative gene cluster and all regulate melanin deposition, their genetic and regulatory relationships were further analyzed. When induced to produce melanin on BMM medium, the single-deletion mutants of *VdZFP1* or *VdZFP2* did not affect the expression of *VdPKS9*, but the expression level was significantly downregulated in the double-deletion mutant (Fig. 3A). Meanwhile, the expression levels of *VdZFP1* and *VdZFP2* in the *VdPKS9* deletion mutant (Δ*VdPKS9*) were consistent with that of the WT strain (Fig. 3B). Surprisingly, *VdZFP1* and *VdZFP2* showed opposite expression patterns compared to *VdCmr1* in the *VdPKS9* overexpression strain (OE^*VdPKS9*^) (Fig. 3B). All these results contradicted the role of PKS9 in melanin deposition in the *VdPKS9* deletion mutant and formation of the hyphae-type strain mediated by overexpressing *VdPKS9*. These results suggest that there were no direct regulatory relationships between VdZFP1 or VdZFP2 and *VdPKS9*, and they may not form a typical *PKS* gene cluster.

To determine the genetic relationships of *VdZFP1* and *VdZFP2* with *VdPKS9*, *VdPKS9* was deleted in the *VdZFP1* or *VdZFP2* mutant background (Δ*VdZFP1_ VdPKS9* and Δ*VdZFP2_ VdPKS9*). Examination of colony phenotypes on PDA medium after 7 days revealed that the absence of *VdPKS9* restored the defects in melanin production of *VdZFP1* and *VdZFP2* mutants (Fig. 3C). Gene expression analysis showed that the deletion of *VdPKS9* in the *VdZFP1* and *VdZFP2* double mutant background resulted in significantly increased expression levels of melanin biosynthetic genes compared to either the *VdZFP1* or *VdZFP2* mutants (Fig. 3D, E). In addition, overexpression of *VdPKS9* in either the *VdZFP1* or *VdZFP2* mutant promoted the growth of aerial hyphae and the formation of albino colonies (Additional file 1: Figure S4). These results indicated that the defective melanin deposition in *VdZFP1* or *VdZFP2* mutant had no direct connection with *VdPKS9*, and also confirmed that VdZFP1 and VdZFP2 are not directly regulated by VdPKS9*.*

### VdZFP1 and VdZFP2 regulate the development of microsclerotia

**Fig. 4:**
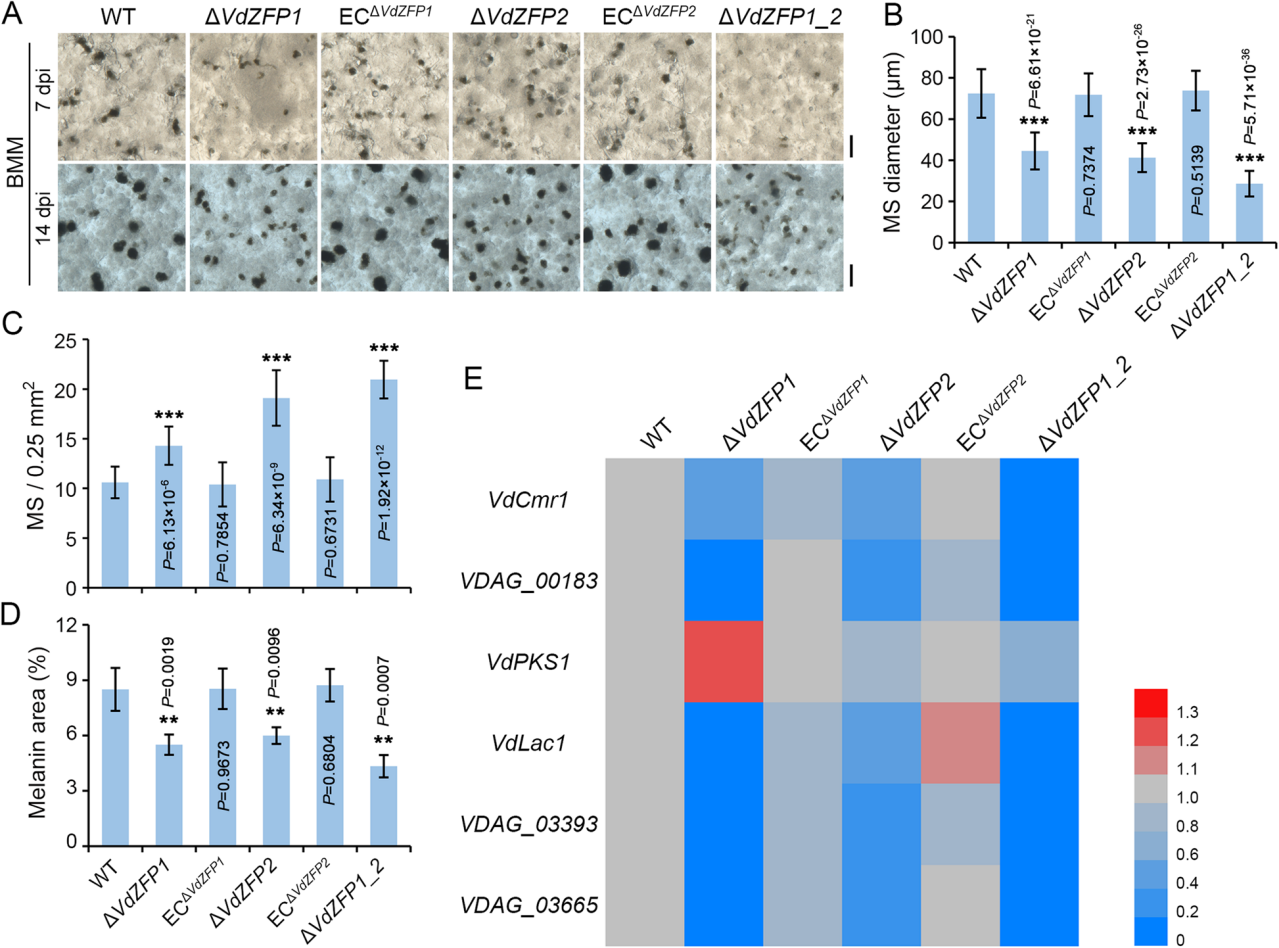
VdZFP1 and VdZFP2 participate in microsclerotia development of *Verticillium dahliae*. **A** Microsclerotia morphology of WT, Δ*VdZFP1*, EC^Δ*VdZFP1*^, Δ*VdZFP2*, EC^Δ*VdZFP1*^, and Δ*VdZFP1_2* strains. Each strain was cultured on BMM plates covered with cellophane membranes. The development of microsclerotia was observed at 7 and 14 dpi after incubation at 25 °C in the dark and photographed with a stereoscope. Each strain was repeated three times independently, and at least three plates were observed each time. Scale bar = 100 μm. **B–D** Statistics on differences in the number, volume, and melanin coverage of microsclerotia between WT, deleted mutants, and complemented strains. WT, Δ*VdZFP1*, EC^Δ*VdZFP1*^, Δ*VdZFP2*, EC^Δ*VdZFP1*^, and Δ*VdZFP1_2* strains were cultured on BMM medium at 25 °C in the dark. After incubating for 7 days, the diameter of 30 matured microsclerotia of each strain was measured, while the number or melanin coverage of microsclerotia was calculated from 20 or 6 visual fields of 0.5 or 1 mm squared, respectively. **B** Microsclerotia diameter, **C** microsclerotia number, and** D** melanin coverage of microsclerotia. Error bars represent the standard deviation of each independent experiment, and all experiments performed three repeats, ^**^P < 0.01, and ^***^P < 0.001 (Student’s *t* test). **E** Analyses of the relative expression of melanin biosynthesis genes during the microsclerotia development among WT, Δ*VdZFP1*, EC^Δ*VdZFP1*^, Δ*VdZFP2*, EC^Δ*VdZFP1*^, and Δ*VdZFP1_2* strains. All strains were grown on BMM medium at 25 °C in the dark and collected at 7 dpi. Compared with WT strain, the expression level of each gene in transformants was detected by RT-qPCR for 3 repetitions using the 2^−ΔΔCT^ method and finally presented as a heatmap. This experiment was independently repeated 3 times to determine the trend

Since increased melanin biosynthesis is tightly coupled to microsclerotia development and stress responses [40, 59], defective melanin deposition in the *VdZFP1* and *VdZFP2* deletion mutants pointed to the possibility that VdZFP1 and VdZFP2 regulate the development of microsclerotia. To analyze this further, the microsclerotia from *VdZFP1* and *VdZFP*2 deletion strains were investigated for morphological differences, enumerated, and assessed for melanin accumulation. All strains were induced to produce microsclerotia on both BMM medium covered with cellophane membranes and observed under a stereomicroscope. At 7 dpi, the microsclerotia of the deletion mutants showed differences relative to the WT and complemented strains in size, melanization, and morphology. Specifically, the *VdZFP1* and *VdZFP*2 mutants produced more (increased by approximately 30 to 70%) and smaller (about 60% the size of the WT strain) microsclerotia, and the exhibited reduced accumulation of melanin (Fig. 4A–D). Furthermore, the abnormal microsclerotia development (double the quantity and halve the volume) and the decrease in melanin accumulation (about 50%) were more obvious in the *VdZFP1/VdZFP2* double-deletion strain, and most microsclerotia remained as melanized but unaggregated swollen precursors (Fig. 4A–D). These defects persisted, except for an increase in volume, number, and pigmentation in all mutants until 14 dpi, whereas complemented strains basically rescued these defects (Fig. 4A). Consequently, these results indicated that VdZFP1 and VdZFP2 positively regulate melanin production as well as the development and maturation of microsclerotia in *V. dahliae*.

As microsclerotia were progressively enriched for melanin in *V. dahliae*, the relative expression of melanin biosynthesis genes was further analyzed. Quantitative results showed that the expression levels of these genes were consistent with the developmental status of microsclerotia, and most of the detected genes were downregulated in *VdZFP1* or/and *VdZFP2* mutants (Fig. 4E). Moreover, the *VdZFP1/VdZFP2* double-deletion mutant produced more melanin-deficient microsclerotia compared to the wild type, suggesting that VdZFP1 and VdZFP2 are not unique regulators of these processes. Overall, these results demonstrated that the development of microsclerotia is synchronous with the deposition of melanin and that VdZFP1 and VdZFP2 are crucial to regulate the development and melanization of microsclerotia in *V. dahliae*.

### VdZFP1 and VdZFP2 positively impact the microsclerotia maturation by regulating melanin biosynthesis directly

**Fig. 5:**
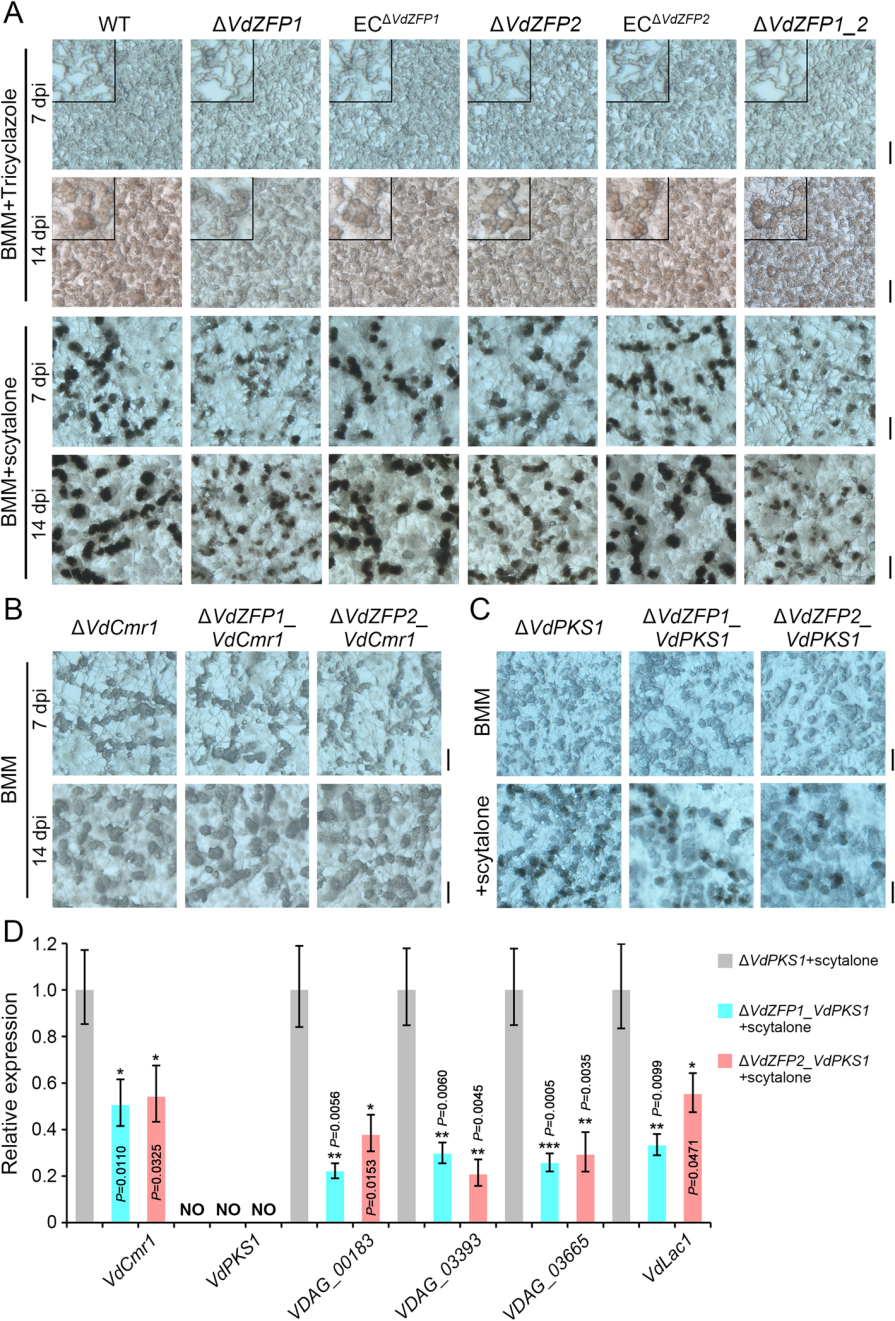
*Verticillium dahliae* VdZFP1 and VdZFP2 regulate the melanization of microsclerotia. **A** Microsclerotia morphology of the WT, Δ*VdZFP1*, EC^Δ*VdZFP1*^, Δ*VdZFP2*, EC^Δ*VdZFP1*^, and Δ*VdZFP1_2* strains exposed to the melanin biosynthesis pathway inhibitor tricyclazole and intermediate scytalone. All of the indicated strains were cultured on the BMM medium or supplemented with the tricyclazole (60 mg/mL) and scytalone (50 mg/mL) for 7 and 14 days. The microsclerotia were observed using a stereoscope, scale bar = 100 μm. **B** Microsclerotia phenotypes of albino Δ*VdCmr1* and double-deleted mutants (Δ*VdZFP1_VdCmr1* and Δ*VdZFP2_VdCmr1*). The microsclerotia were inducted on BMM medium for 7 and 14 days and were observed using a stereoscope, scale bar = 100 μm. **C** Investigation of microsclerotia melanization in Δ*VdPKS1* and double-deleted mutants (Δ*VdZFP1_VdPKS1* and Δ*VdZFP2_VdPKS1*) supplemented with the melanin intermediate scytalone. The microsclerotia were inducted on normal or scytalone (50 mg/mL) supplemented BMM medium for 7 days and were observed using a stereoscope, scale bar = 100 μm. **D** Relative expression analyses of the melanin biosynthesis genes of the indicated strains in **C** following supplementation with scytalone. The strains cultured on BMM medium contained 50 mg/mL scytalone for 7 days were collected from above (**C**) experiment. Using Δ*VdPKS1* as control, the expression levels of each gene in mutants were detected by RT-qPCR for 3 repetitions using the 2^−ΔΔCT^ method. Error bars represent standard errors of the mean, and ^*^P < 0.05, ^**^P < 0.01, and ^***^P < 0.001 (one-way ANOVA). All experiments were independently repeated 3 times

To analyze the effects of VdZFP1 and VdZFP2 on the development of microsclerotia, the melanin biosynthesis inhibitor tricyclazole and the DHN-melanin pathway intermediate scytalone [1, 60] were additionally supplied into BMM medium. Tricyclazole (60 μg/mL) treatment resulted in the loss of melanin deposition in all strains, but the compact and swollen precursor structures still formed at 7 and 14 dpi (Fig. 5A). Meanwhile, tricyclazole was replaced by scytalone (50 μg/mL) to evaluate the efficiency of melanin biosynthesis in each strain. Although melanin accumulation and development of microsclerotia in *VdZFP1* and *VdZFP*2 mutants were prevalent in both, their numbers and size were reduced relative to the WT and complemented strains (Fig. 5A). These results suggested that although VdZFP1 and VdZFP2 are not directly involved in the formation of microsclerotia precursors, they have a role in the regulation of melanin biosynthesis during the maturation of microsclerotia in *V. dahliae*.

Since VdZFP1 and VdZFP2 also regulate the size of microsclerotia (Fig. 4B), we further aimed to distinguish their roles in regulating the formation of microsclerotia and melanin biosynthesis. Two key melanin biosynthesis factors, *VdCmr1* and *VdPKS1*, were deleted in both the *VdZFP1*- and *VdZFP*2-deletion mutant strains (Δ*VdZFP1_VdCmr1*, Δ*VdZFP2_VdCmr1*, Δ*VdZFP1_VdPKS1*, and Δ*VdZFP2_VdPKS1*). Interestingly, all the albino mutants displayed the similar melanization-defective microsclerotia (Fig. 5B, C), indicating that VdZFP1 and VdZFP2 regulate melanin biosynthesis rather than direct microsclerotia formation in *V. dahliae*. To further determine the regulation of melanin biosynthesis by VdZFP1 and VdZFP2 during the development of microsclerotia, cultures supplemented with the melanin intermediate scytalone was investigated. Expectedly, both *VdPKS1* and *VdPKS1* double-deletion mutants recovered the ability to accumulate melanin and form melanized microsclerotia on medium supplemented with scytalone (50 μg/mL) (Fig. 5C; Additional file 1: Figure S5). Compared to *VdPKS1* mutants, the expression of genes related to melanin biosynthesis were expressed at low levels in *VdPKS1* double-deletion mutants cultured on scytalone-supplemented medium (Fig. 5D). Thus, VdZFP1 and VdZFP2 participate in melanin biosynthesis but not microsclerotia formation directly by positively regulating the *VdPKS1* cluster and its regulatory gene *VdCmr1* in *V. dahliae*.

### VdZFP1 and VdZFP2 interact with VdCmr1 to regulate melanin biosynthesis

**Fig. 6:**
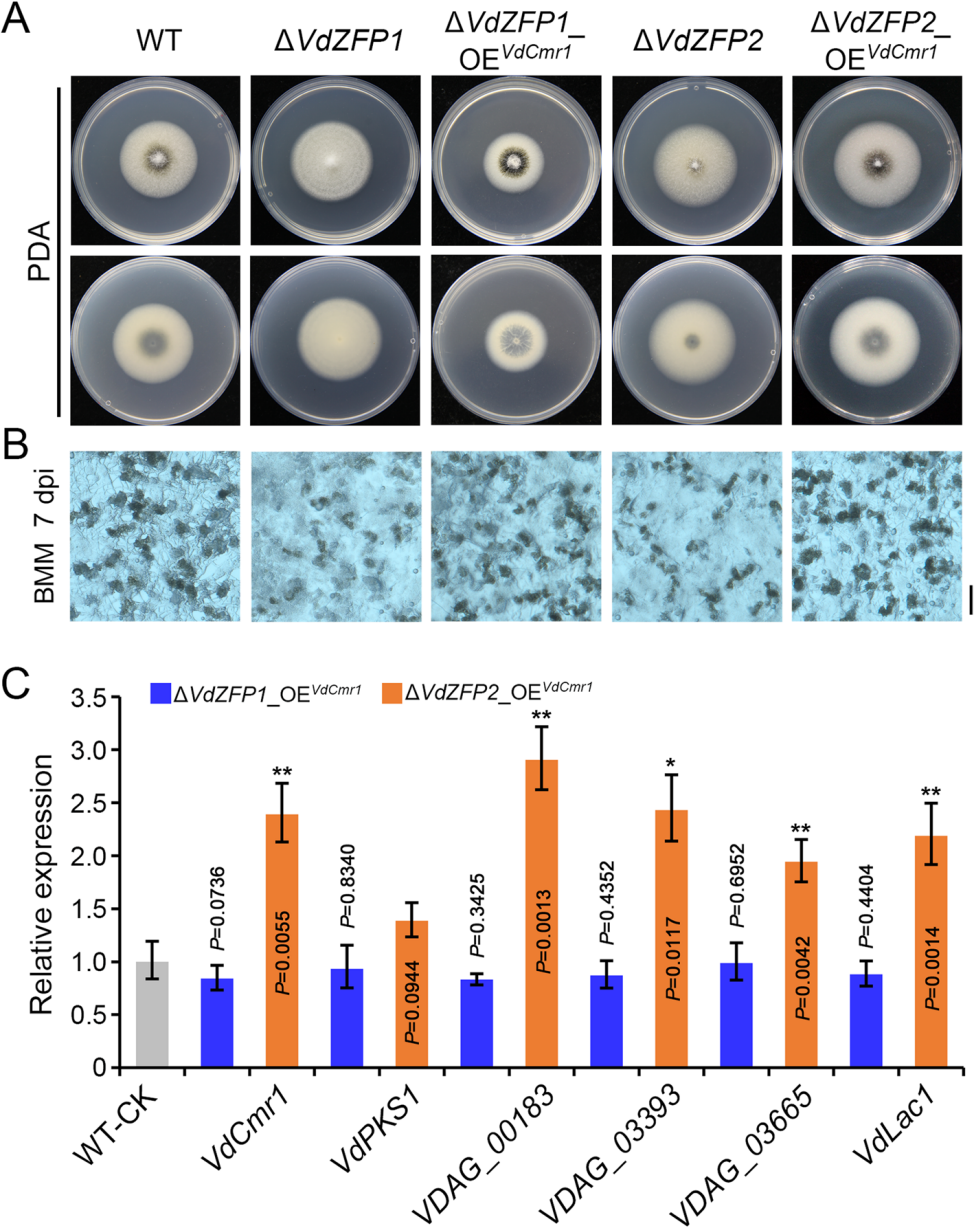
VdZFP1 and VdZFP2 mediate melanin deposition of microsclerotia by positively controlling *VdCmr1* in *Verticillium dahliae*. **A** Colony morphology of the WT strain, mutants (Δ*VdZFP1* and Δ*VdZFP2*), and *VdCmr1*-overexpressed strain (Δ*VdZFP1_*OE^*VdCmr1*^ and Δ*VdZFP2_*OE^*VdCmr1*^). The indicated strains were cultured on PDA medium at 25 °C in the dark and were photographed 7 days after incubation. Each strain was inoculated at least three plates. **B** Microsclerotia morphology of indicated strains. The microsclerotia development of each strain was observed at 7 dpi after incubating on the BMM plates covered with cellophane membranes at 25 °C in the dark and photographed with a stereoscope. Each strain was repeated three times independently, and at least three plates were observed each time. Scale bar = 100 μm. **C** Relative expression analyses of melanin-related genes in indicated strains. The strains were grown on BMM medium at 25 °C in the dark and were collected at 7 dpi for RT-qPCR. The results of 3 repetitions analyzed by the 2^−ΔΔCT^ method with WT strain as control. This experiment was independently repeated 3 times to determine the trend. Error bars represent standard errors of the mean, and ^**^P < 0.01, and.^***^P < 0.001 (one-way ANOVA)

Similar to other melanin-producing filamentous fungi, *V. dahliae* also relies on the Cmr1p-like transcription factor VdCmr1 to regulate the *VdPKS1* gene cluster in melanin biosynthesis [9]. Gene expression analysis showed that *VdCmr1* was significantly downregulated in *VdZFP1* and *VdZFP*2 mutants in the Δ*VdPKS1* background not only during microsclerotia induction but also in response to scytalone treatment (Figs. 4E and 5D). To demonstrate the regulatory relationship between the two ZFPs and VdCmr1, the expression of *VdZFP1* and *VdZFP*2 were examined in the *VdCmr1* mutant (Δ*VdCmr1*). There was no difference in the expression of the *VdZFP1* and *VdZFP*2 between the *VdCmr1* mutant and the WT strain (Additional file 1: Figure S6A). This one-way regulatory relationship suggested that *VdZFP1* and *VdZFP*2 may play roles upstream of *VdCmr1*. *VdCmr1*-overexpressing strains were generated in the *VdZFP1* and *VdZFP*2 single-deletion background (Δ*VdZFP1*_OE^*VdCmr1*^ and Δ*VdZFP2*_OE^*VdCmr1*^; Additional file 1: Figure S6B) to solidify this result. As expected, the overexpression of *VdCmr1* recovered the melanin defects in each of the *VdZFP1* and *VdZFP*2 single-deletion mutants on PDA medium at 7 dpi (Fig. 6A). The *VdCmr1*-overexpressing strains also exhibited microsclerotia morphology consistent with the WT strain on BMM medium at 7 and 14 dpi (Fig. 6B and Additional file 1: Figure S6C). Moreover, when *VdCmr1* was overexpressed in each of the *VdZFP1* and *VdZFP2* mutants, the downstream genes controlled by *VdCmr1* approached or exceeded the expression levels of WT strain (Fig. 6C). These results suggested that VdCmr1 is a target for VdZFP1 and VdZFP2 to regulate the melanin biosynthesis pathway in *V. dahliae*.

To detect whether VdZFP1 and VdZFP2 act as TFs to regulate *VdCmr1*, their activation and promoter binding functions were tested. The yeast cells transformed by BD-VdZFP1 or BD-VdZFP2 recombinant plasmids can grow on (SD)-Trp-His plates, indicating that VdZFP1 and VdZFP2 have transcriptional activation functions (Additional file 1: Figure S6D). However, the results of yeast one-hybrid (Y1H) system showed that VdZFP1 or VdZFP2 cannot regulate VdCmr1 by binding to its promoter (Additional file 1: Figure S6E). These results suggested that VdZFP1 and VdZFP2 do not act directly as TFs to regulate *VdCmr1* during melanin biosynthesis.

**Fig. 7:**
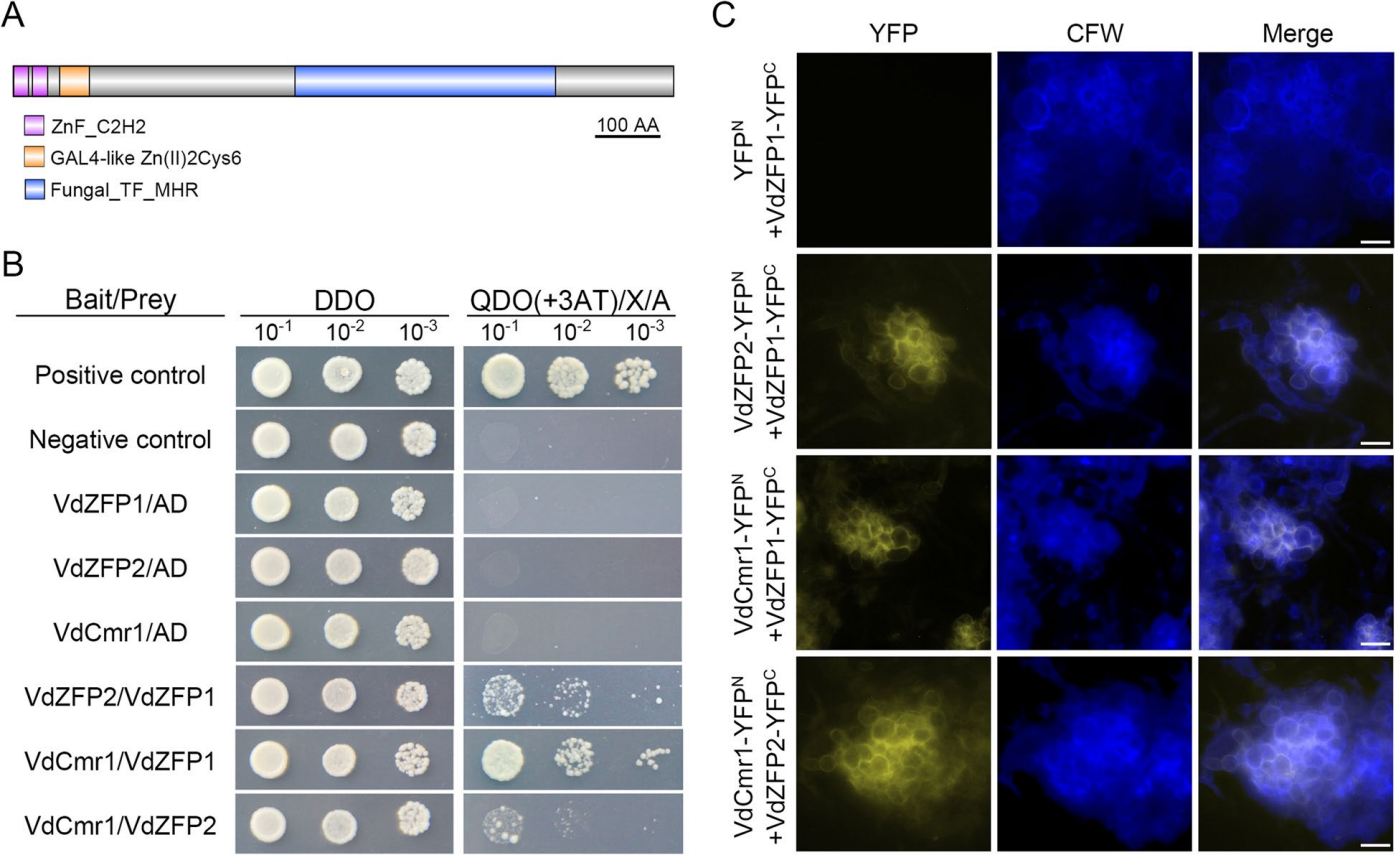
*Verticillium dahliae* VdZFP1, VdZFP2, and VdCmr1 interact directly with each other on the cell wall of microsclerotia precursors. **A** The conserved domains of VdCmr1 in *V. dahliae*. The protein sequence of VdCmr1 was predicted by SMART, InterPro, and Pfam and each domain was labeled in different colors. Scale bar = 100 amino acids. **B** Interaction analyses among VdZFP1, VdZFP2, and VdCmr1 in a yeast two-hybrid system. The CDS regions of *VdZFP1*, *VdZFP2*, and *VdCmr1* were linked into pGADT7 and pGBKT7 vectors to obtain the prey and bait constructs. Each bait construct was co-transformed with pGADT7 vector into yeast cells to detect self-activation, while the yeast cells containing the bait and prey constructs were used to detect interaction. Yeast cells with tenfold serial dilutions were cultured on DDO (SD lacking Leu and Trp) medium and QDO (SD lacking Leu, Trp, His, and Ade) medium supplemented with 3AT (2.5 mM for pGBKT7-VdZFP1 and 70 mM for pGBKT7-VdCmr1), 20 μg/mL X-a-Gal, and 0.1 μg/mL AbA. The yeast cells co-transformed with pGADT7-T and pGBKT7-53 or pGBKT7-Lam vectors were set as the positive or negative control. The Interaction phenotypes were photographed at 5 dpi. This experiment was repeated 3 times. **C** Bimolecular fluorescence complementation assays among VdZFP1, VdZFP2, and VdCmr1. The recombinant plasmids of VdZFP1, VdZFP2, and VdCmr1 fused with C- or N-terminal of YFP were paired and co-transformed into WT strain. The recombinant plasmids co-transformed with YFP^C^ or YFP^N^ were negative controls. The CFW dye was used as the cell wall marker. The CFW and YFP signals were observed by fluorescence microscopy. Scale bars = 20 μm

The C2H2-type zinc fingers and Zn(II)2Cys6 binuclear cluster are important for binding to the targets [27]. VdCmr1 was predicted to be a classic TF containing two C2H2-type zinc fingers, a GAL4-like Zn(II)2Cys6 binuclear cluster DNA-binding domain and a fungal transcription factor regulatory middle homology region (Fig. 7A), implying that VdZFP1, VdZFP2, and VdCmr1 may interact with each other in regulating the microsclerotia melanization of *V. dahliae*. To test this possibility, the interaction relationships among three proteins were examined using a yeast two-hybrid (Y2H) system. Firstly, the self-activation of BD-VdZFP1, BD-VdZFP2, and BD-VdCmr1 were examined, and each transformed yeast cells can be inhibited unless activation occurs on synthetic dropout (SD)-Trp-Leu-His-Ade (QDO) plates supplemented with different concentrations (2, 10, 50 mM, respectively) of 3AT (3-amino-1,2,4-triazole) (Fig. 7B). The positive and negative controls were introduced, and the interactions were examined using one-to-one Y2H assays. The yeast strains co-transformed with AD-VdZFP1 and BD-VdZFP2, AD-VdZFP1 and BD-VdCmr1 or AD-VdZFP2 and BD-VdCmr1 recombinant plasmids could grow on QDO plates supplemented with 3AT, 0.1 μg/mL AbA (Aureobasidin A), and 20 μg/mL X-α-Gal (5-bromo-4-chloro-3-indolyl-α-D-galactopyranoside) for 5 days and the colonies turned blue (Fig. 7B). These results indicate that VdZFP1, VdZFP2, and VdCmr1 interact with each other directly in the Y2H system.

To further determine the interaction relationships between VdZFP1, VdZFP2, and VdCmr1, bimolecular fluorescence complementation (BiFC) assays for observation of yellow fluorescent protein (YFP) signals were performed by co-expressing VdZFP1-YFP^C^ and VdZFP2-YFP^N^, VdZFP1-YFP^C^ and VdCmr1-YFP^N^, or VdZFP2-YFP^C^, and VdCmr1-YFP^N^ recombinant plasmids in WT strain. Co-expression of VdZFP1-YFP^C^ and YFP^N^ was treated as negative control (the data of co-expression of VdZFP2-YFP^C^ and YFP^N^, VdZFP2-YFP^N^ and YFP^C^, or VdCmr1-YFP^N^ and YFP^C^ are not shown). Interestingly, the YFP signals were observed on the cell wall of aggregated swollen precursors without melanin accumulation in strains co-expressed with VdZFP1-YFP^C^ and VdZFP2-YFP^N^, VdZFP1-YFP^C^ and VdCmr1-YFP^N^, and VdZFP2-YFP^C^ and VdCmr1-YFP^N^ (Fig. 7C), while no yellow fluorescence was observed in the negative control, indicating that VdZFP1, VdZFP2, and VdCmr1 interact with each other on the cell walls of immature microsclerotia. The colocalization of YFP and CFW signals further confirmed that the interactions among them occur on the cell wall (Fig. 7C). Together, these results demonstrated that melanin biosynthesis related to microsclerotia development in *V. dahliae* relies on the interaction between VdCmr1, VdZFP1, and VdZFP2.

### VdZFP1 and VdZFP2 are necessary for abiotic stress tolerance rather than pathogenicity

**Fig. 8:**
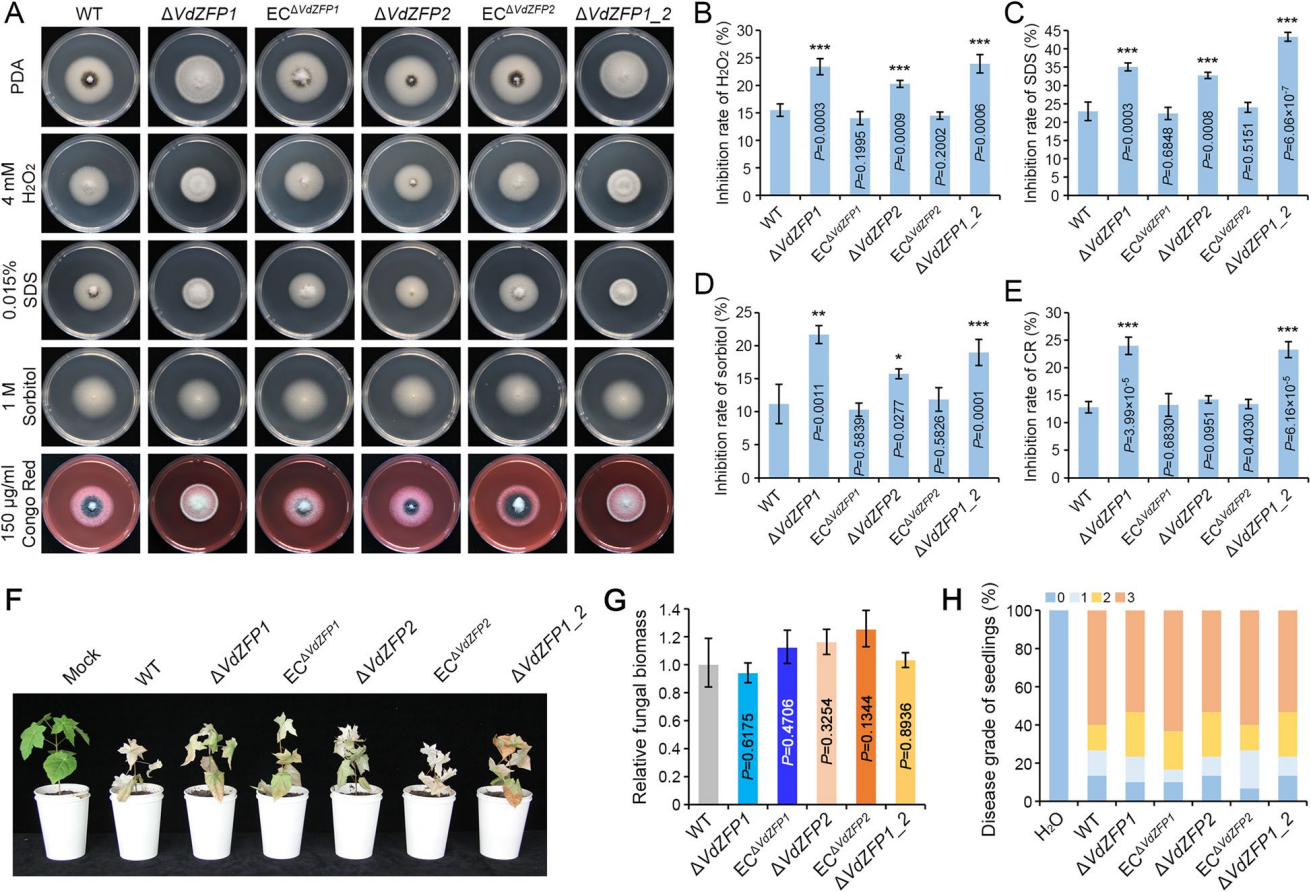
VdZFP1 and VdZFP2 contribute to the response of *Verticillium dahliae* to environmental stresses rather than pathogenicity. **A** Colony morphology of WT, Δ*VdZFP1*, EC^Δ*VdZFP1*^, Δ*VdZFP2*, EC^Δ*VdZFP1*^, and Δ*VdZFP1_2* strains growth on medium supplemented with various stressors. These strains grown on PDA medium that supplemented with 4 mM H_2_O_2_, 0.015% SDS, 1 M sorbitol, and 150 μg/mL Congo red. The phenotypes were photographed 7 days after incubation at 25 °C in the dark. Each strain was inoculated at least 3 plates and repeated 3 times independently. **B–E** Sensitivity of indicated strains to stress factors. The inhibition rate of indicated strains responsible for various stressors in panel **A**. Error bars are standard errors calculated from six replicates, and this experiment performed three repeats. ^*^P < 0.05, ^**^P < 0.01 (Student’s *t* test). Sensitivity of indicated strains to **B** H_2_O_2_, **C** SDS, **D** sorbitol, and **E** Congo red. **F** Pathogenicity assay of the indicated strains on shantung maple. Shantung maple seedlings were inoculated with WT, Δ*VdZFP1*, EC^Δ*VdZFP1*^, Δ*VdZFP2*, EC^Δ*VdZFP1*^, and Δ*VdZFP1_2* strains, while the H_2_O treatment was set as negative control. The Verticillium wilt symptom were photographed at 50 dpi. **G** Quantification of the fungal biomass in maple stems by qPCR following inoculation of the indicated strains. Samples were collected from the stem base of infected plants at 50 dpi. The *At18S* gene of shantung maple was served as an endogenous control to evaluate the endophytic colonization of *V. dahliae* by quantifying *VdEF-1α*. The pathogenicity was analyzed with three replicates of 10 3-month-old maple trees, and the fungal biomass was calculated by three independent biological replicates. Error bars represent standard errors. *P* > 0.05 means not significant (one-way ANOVA). **H** Evaluation of disease grade of shantung maple seedlings. The disease grade was divided based on the Verticillium wilt symptoms and analyzed with three replicates of maple pathogenicity tests

Melanin is considered to be a physical barrier to maintain homeostasis of fungi, of course, it is also crucial for *V. dahliae* to resist adverse environmental factors and long-term survival [60]. To evaluate the abiotic stress response of *VdZFP1* and *VdZFP2*, *VdZFP1*, and/or *VdZFP2* mutants, WT and complemented strains were cultured on the PDA medium supplemented with 4 mM hydrogen peroxide (H_2_O_2_), 0.015% sodium dodecyl sulfate (SDS), 1 M sorbitol, and 150 μg/mL congo red for 7 days, respectively. The inhibition rate of each strain from different stresses revealed that the sensitivities of *VdZFP1* and *VdZFP2* mutants to oxidative, osmotic, and cell wall integrity stresses were significantly increased (Fig. 8A–E). This suggested that both VdZFP1 and VdZFP2 are necessary for *V. dahliae* to resist abiotic stresses.

Mutants of *V. dahliae* with deficient melanin and dysplastic microsclerotia are often accompanied by weak pathogenicity, such as Δ*VdHog1* and Δ*VdMsb* [38, 61]. To verify the contribution of VdZFP1 and VdZFP2 to virulence, all strains were inoculated into 3-month-old Shantung maple seedlings. The Verticillium wilt symptoms and pathogen biomass of *VdZFP1* and *VdZFP2*, and *VdZFP1/VdZFP2* double-deletion mutants were similar to those of WT and complemented strains at 50 dpi (Fig. 8F, G). The disease severity levels further revealed that the mortality rate of each strain was above 50% (Fig. 8H). In addition, the pathogenicity phenotype and pathogen biomass of *VdZFP1* and *VdZFP2* mutants on cotton and tobacco were also unchanged (Additional file 1: Figure S7), which indicated that VdZFP1 and VdZFP2 are not pathogenicity factors in *V. dahliae*.

Gene synteny analysis of the neighboring protein-coding genes of *VdZFP1* and *VdZFP2* in *V. dahliae* and their homologs in *C. gloeosporioides* showed they share a conserved fragment composed of 11 (or 10) genes (Additional file 1: Figure S8). The two homologs *CgZFP1* and *CgZFP2* were not essential for melanization of appressoria in *C. gloeosporioides*, but the *CgZFP2* mutant decreased radial growth and lost pathogenicity on *Liriodendron chinense*. *CgZFP1* had no impact on the radial growth or pathogenicity*.* (Additional file 1: Figure S9). These results indicate that the functions of VdZFP1 and VdZFP2 are not conserved in these phytopathogenic fungi.

## Discussion

**Fig. 9:**
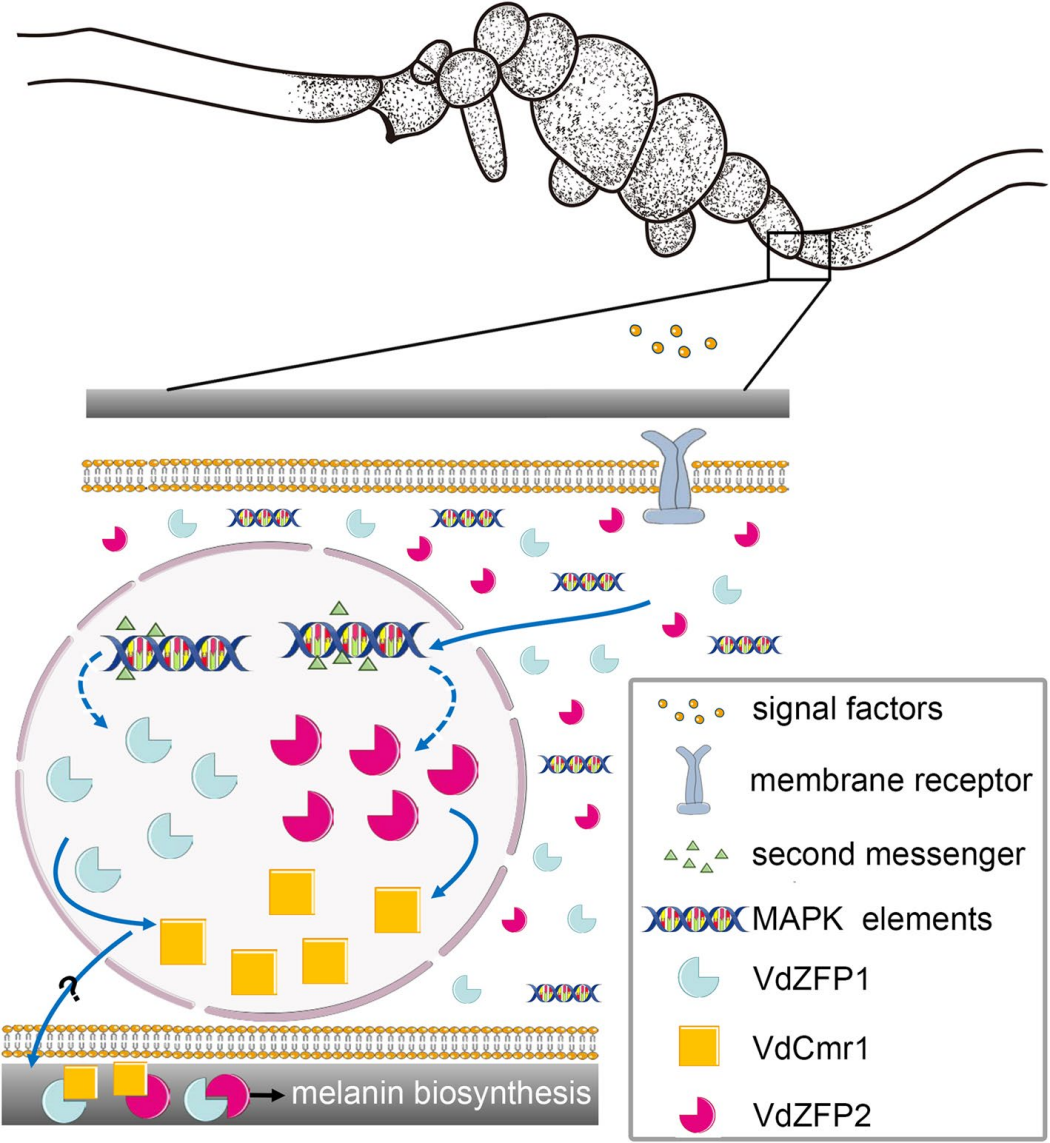
A working model of VdZFP1 and VdZFP2 during microsclerotia development of *Verticillium dahliae*. The cytoplasm-localized C2H2-type zinc finger proteins VdZFP1 and VdZFP2 enter the nucleus by sensing environmental signals and are regulated by the MAPK signaling pathway to promote microsclerotia development and melanization by interacting with VdCmr1 on the cell wall

ZFPs are the largest and most diverse family of TFs in eukaryotic genomes [62]. They play important roles in various molecular processes, such as replication, repair, transcription, translation, metabolism, signaling, cell proliferation, apoptosis, targeted DNA or RNA recognition and protein–protein interactions [63, 64]. In phytopathogenic fungi, ZFPs contribute to regulation of responses to a range of environmental factors and stressors, secondary metabolism, growth, and virulence [65–67]. The differences in the conserved zinc finger motifs and DNA-binding domains, this protein family has been classified into several types, while the C2H2 type is the most abundant and well-studied [68]. In *V. dahliae*, more than 90 annotated C2H2-type ZFPs (79 were typical) consist of the second largest superfamily following the Zn(II)2Cys6-type TFs [67, 69], and some of them were further predicted to respond to host induction, microsclerotia development, and starvation stress of *V. dahliae*. However, the vast majority of ZFPs have not been characterized. In this study, we identified two ZFPs (VdZFP1 and VdZFP2) involved in stress tolerance but not in pathogenicity. Further, VdZFP1 and VdZFP2 positively promote melanin biosynthesis during microsclerotia maturation by interacting with VdCmr1 to regulate the *VdPKS1*-cluster contributing to DHN-melanin production in *V. dahliae* (Fig. 9).

So far, the regulatory network of melanin production is largely unknown in *V. dahliae*, even if the biochemical pathway and many of the enzymes involved in its synthesis are well defined [60]. Various TFs function in the modulation of melanin biosynthesis and also have established functional roles in host epidermal penetration, growth, stress resistance, and virulence in *V. dahliae* [48, 52, 57, 58]. VdZFP1 and VdZFP2, located upstream of putative *VdPKS9* gene cluster (Fig. 1A) and the colony phenotypes of *VdZFP1* and *VdZFP2* mutants, showed serious melanin deficiencies on PDA medium (Fig. 2A), which naturally led us to associate their regulatory function with VdPKS9. Previously, we described the morphology-differentiation switch of VdPKS9 in *V. dahliae* that negatively regulates microsclerotia development and melanin biosynthesis while promoting hyphal growth [39]. However, these opposing phenotypes are also interconnected. On the one hand, the deletion of *VdZFP1* or *VdZFP2* did not alter the expression of *VdPKS9* and its ability to negatively regulate melanin biosynthesis (Fig. 3 and Additional file 1: Figure S4), which ruled out the possibility of VdZFP1 and VdZFP2-mediated melanin biosynthesis by regulating *VdPKS9*. On the other hand, *VdZFP1/VdZFP2* double-deletion mutants still deposited melanin in microsclerotia (Fig. 4A) and may be due to the accumulation of melanin caused by downregulation of *VdPKS9* (Fig. 3A). Moreover, the consistent upregulation of VdZFP1 and VdZFP2 in *VdPKS9*-overexpressed strain (Fig. 3B) may be the reason for the production of melanin by hyphae-type strains after long-term induction on BMM medium [39]. These results suggest that prolonged growth and induction may partly counteract the conflicts between the gene expression of *VdZFPs* and *VdPKS9*, and thus melanin biosynthesis in *V. dahliae* is not reliant on VdZFP1 and VdZFP2. In addition to regulating melanin biosynthesis, the different colony diameters of Δ*VdZFP1* (Fig. 2A, B; Additional file 1: Figure S3) and Δ*VdZFP1_*OE^*VdCmr1*^ or Δ*VdZFP1_*OE^*VdPKS9*^ (Figs. 3A and 6A and Additional file 1: Figure S4) imply that VdZFP1 is also involved in growth and in responses to environmental cures and stressors.

The mechanisms of microsclerotia formation and melanization in *V. dahliae* have come under increased scrutiny since melanized microsclerotia are critical to its disease cycle as survival structures and primary inoculation [9, 38, 43, 70]. In this study, the expression of both *VdZFP1* and *VdZFP2* was elevated in response to the induction of microsclerotia development (Fig. 2D; Additional file 1: Figure S2B) and localized in swollen hyphae (Fig. 1C); thus, we focused on the potential functional roles of VdZFP1 and VdZFP2 in development and melanization of microsclerotia. Deletion of *VdZFP1* and/or *VdZFP2* leads to the formation of a larger number of severely developmentally delayed microsclerotia (Fig. 4A–D). Although limited evidence suggests that a few transcriptional regulators such as Vst1 and VdAda1 are involved in this process [54, 71], the direct regulator of microsclerotia formation has not been discovered. Nearly all abnormalities in microsclerotia development are accompanied by changes in melanin production, though microsclerotia development and melanin production can be uncoupled [9, 40, 59]. The downregulated expression levels of melanin biosynthetic genes in the *VdZFP1* and *VdZFP2* mutants (Fig. 4E) indicated that the microsclerotia development regulated by VdZFP1 and VdZFP2 was also linked to the regulation of melanin biosynthesis. Thus far, all the analyzed ZFPs in *V. dahliae*, including conserved VdCrz1 and VdCmr1, are involved in the regulation of the development of melanized microsclerotia. For example, the deletion of VdMsn2 or VdCf2 generate excessively melanized and denser microsclerotia, while the Zn(II)2Cys6-type TF Vdpf negatively regulates these processes [47, 51, 67]. These findings coupled with melanin deposition in the *VdZFP1*/*VdZFP2* double-deletion mutant (Figs. 3A and 4A), albeit at reduced levels, suggest that other ZFPs or TFs may regulate this process. Thus, the melanization of microsclerotia is extremely complex, and the regulatory role of the ZFPs in *V. dahliae* requires further exploration.

Besides VdPKS9, another key regulator of melanin biosynthesis in *V. dahliae* is VdCmr1 [9, 39]. As demonstrated herein, VdZFP1 and VdZFP2 act epistatic to VdCmr1 as positive regulators of melanin biosynthesis and microsclerotia development (Fig. 6; Additional file 1: Figure S6). Zinc fingers are short motifs composed of two or three β-layers and one α-helix. The number and structural differences in zinc fingers in one ZFP enable variability in the binding specificity of targets [72, 73]. Coincidentally, VdCmr1 contains two C2H2-type and one Zn(II)2Cys6-type zinc finger motifs (Fig. 7A), which implied that VdZFP1 and VdZFP2 may interact with VdCmr1. Further, we determined by Y2H and BiFC assays that VdZFP1, VdZFP2, and VdCmr1 interact on the cell walls of immature microsclerotia where there is active melanin deposition (Fig. 7B, C). In contrast to TFs that may have a stable nuclear localization, we observed the transfer of fluorescence signals from the cytoplasm of swollen hyphae and germinating conidia to the nucleus by introducing osmotic stress (Fig. 1C), a factor that affects microsclerotia development [57]. This is reminiscent of VdCrz1, which is cytoplasmic until signals from high concentrations of extracellular calcium ions trigger its localization into the nucleus [21]. In eukaryotes, the translocation and reversible localization of signaling proteins are common, especially the nuclear localization of MAPK pathway elements [45, 46]. The HOG-MAPK pathway and its upstream or downstream elements transduce the signals in response to osmotic stress and for *VdCmr1*-mediated melanin biosynthesis and the development of melanized microsclerotia in *V. dahliae* [9, 39, 47, 48, 74, 75]. The RT-qPCR results of mutants of the starvation signaling and osmotic stress response-related VdHog1 pathway indicate that VdZFP1 and VdZFP2 are downstream of VdHog1 (Additional file 1: Figure S10). These results confirm that VdZFP1 and VdZFP2 are located downstream of the HOG-MAPK pathway to regulate the VdCmr1-mediated melanin biosynthesis cluster. Moreover, downstream members of the Cmr1p-mediated pathway catalyze the biosynthesis of melanin on the cell wall of *B. cinerea* [11]. In *V. dahliae*, various environmental factors stimulate the formation and melanization of microsclerotia, and thus the variable subcellular localization of VdZFP1 and VdZFP2, their interactions with VdCmr1 on cell wall, and their ability to regulate melanin biosynthesis are especially interesting.

The linkages between melanin, microsclerotia, and pathogenicity are unresolved [58, 70, 76], though melanin itself is not required for pathogenicity in *V. dahliae* [9]. Melanin in plant pathogenic fungi endows them with several functions such as survival (e.g., in dormant structures), stress tolerance (e.g., in dormant structures or conidia), host invasion (e.g., in appressoria or hyphopodia), and escape host attack (e.g., in conidia) [17, 23, 26, 41, 58, 77], and melanin in variegated structures has its own special regulatory elements, which determines differences in pathogenicity [10, 23, 25]. Our results are consistent with those found for VdCmr1 and VdPKS1 (Fig. 8; Additional file 1: Figure S7), which mediate stress resistance instead of pathogenicity [9, 78]. The phylogenetic analysis indicated that VdZFP1 and VdZFP2 homologs are universal (Fig. 1D) and are adjacent to one another in the genome of *C. gloeosporioides* (Additional file 1: Figure S8). However, functional analysis of their counterparts in *V. dahliae* revealed that the roles of the homologs in vegetative growth, melanin biosynthesis, and pathogenicity to be nearly opposite to those in *C. gloeosporioides* (Additional file 1: Figure S9). Moreover, the interactive and functional similarities of ZFPs cannot be directly inferred by homology, because the diversity of amino acid residues localized in the N-terminal region of the α-helix participate in DNA binding of each ZFP [64, 73]. Therefore, the reduced homology (about 44%; Additional file 1: Figure S8) and the differences in localization of melanin deposition may determine functional differences of VdZFP1 and VdZFP2 homologs.

## Conclusions

In conclusion, we identified two novel ZFPs, VdZFP1 and VdZFP2, that are responsible for melanin biosynthesis in *V. dahliae*, and each is important for stress tolerance and microsclerotia maturation via positive regulation and their interactions with VdCmr1 on the cell wall (Fig. 9). This study provides new insights into the complex regulatory networks of melanin biosynthesis and its functions in *V. dahliae*. Furthermore, the roles of VdZFP1 and VdZFP2 homologs are diversified, and identification of these roles may help reveal new targets for the control of this and other phytopathogenic fungi.

## Methods

### Fungal strains and growth conditions

*Verticillium dahliae* strain AT13, isolated from shantung maple with Verticillium wilt [35], was used as the wild-type (WT) strain in this study. The homologs were cloned from *C. gloeosporioides* strain SMCG1#C which was isolated from *Cunninghamia lanceolata* and collected in the laboratory.

The WT strain and mutants produced in this study were incubated on potato dextrose agar (PDA, 200 g potato, 20 g glucose and 15 g agar per liter) medium supplemented with hygromycin (50 μg/mL) or geneticin (50 μg/mL) at 25 °C in the dark. The hyphal block of each strain was cut and shaken in liquid complete medium (CM, 6 g yeast extract, 6 g acid-hydrolyzed casein, 10 g sucrose per liter) for 3 days and were stored at − 80 °C in 25% glycerin.

Analyses of vegetative growth or various stress tolerance were evaluated by culturing strains on PDA, V8 (200 mL V8 vegetable juice, 2 g CaCO_3_, and 15 g agar per liter), and Czapek (2 g NaNO_3_, 1 g K_2_HPO_4_, 0.5 g KCl, 0.5 g MgSO_4_, 0.01 g FeSO_4_, 30 g sucrose, 15 g agar, and water is added up to 1 L; sucrose was replaced by 17 g starch, 10 g pectin, and 10 g sodium carboxymethyl cellulose, respectively) medium at 25 °C in the dark for 7 days. BMM (5 g glucose, 0.2 g NaNO_3_, 0.52 g KCl, 0.52 g MgSO_4_·7H_2_O, 1.52 g KH_2_PO_4_, 3 μM vitamin B1, 0.1 mM vitamin H, 15 g agar, with pH adjusted to 7.5 and water is added up to 1 L) was used for microsclerotia development assays. TB3 (200 g sucrose, 10 g yeast extract, 10 g casein acid hydrolysate, 10 g glucose, 8 g agar, and water is added up to 1 L) medium was incubated with protoplasts of *V. dahliae* to recover the cell wall. The MM and IM medium were used for observing scytalone-induced melanin phenotypes and ATMT (*Agrobactirium tumfacience* mediated-transformant) method respectively, as previously described [79].

### Characterization and phylogenetic analysis

The identification of gene fragments between the two strains was completed based on the National Center for Biological Information (NCBI) database (https://www.ncbi.nlm.nih.gov/). The analyses of the zinc finger motifs, nuclear localization signals, and other domains in VdZFP1, VdZFP2, and their homologs were identified by using online bioinformatics analysis tools such as InterPro (http://www.ebi.ac.uk/interpro/), Pfam (http://pfam.xfam.org/), and SMART (http://smart.embl-heidelberg.de/), the threshold of e-value < 10^−5^.

Gene synteny analysis of the homologs and gene functional annotations were determined in *V. dahliae* and *C. gloeosporioides* according to the functional annotation in the NCBI database. Based on the domain predictions, the zinc finger motifs and nuclear localization signals were drawn by IBS (Illustrator for Biological Sequences). The phylogenetic tree constructed using MEGA 7.0 with NJ method (http://www.megasoftware.net/).

### Gene deletions, mutant complementation, and overexpression mutants

The genomic DNA and RNA required in this study were extracted using a DNA isolation mini kit (Vazyme, Nanjing, China) and RNA kit (Aidlab Biotech, Beijing, China), while RNA was reverse transcribed into cDNA using cDNA synthesis Supermix (TransGen Biotech, Beijing, China). All steps followed the manufacturer’s instructions.

The plasmid pDHt2 [80] containing the hygromycin resistance gene cassette (*hyg*) was used as a vector for generating single-deletion mutants. First, the sequence-specific primer pairs were designed to amplify the 5′- and 3′-flanking regions (0.8–1.5 kb) of the target genes from genomic DNA of the WT strain. Then, the *EcoR*I- or *Xba*I-linearized plasmid and corresponding amplified fragments were inserted by homologous recombination (ClonExpress II One Step Cloning Kit, Vazyme, Nanjing, China). Similarly, the double-deletion vectors were constructed by linking flanking regions to *Xho*I- or *EcoR*I-linearized pCOM vector [80] containing the geneticin resistance cassette (*G418*) in turn. To complement these genes, the *Xba*I/*EcoR*I-digested pCOM vector was fused with fragments including the native promoter (0.8–1.2 kb) and terminator (0.5–0.8 kb) of target genes for constructing the ectopic complementary strains. To generate the overexpression plasmids, the CDS (coding sequence) of target genes were amplified from cDNA of WT strain, and attached with *Xba*I/*Sac*I-digested pCOM-T0161 vector (pCOM vector added with TrpC promoter).

The recombinant plasmids were transferred into Escherichia coli DH5α competent cells and screened under kanamycin resistance for obtaining positive clones. Subsequently, the recombinant plasmids were transformed into *Agrobacterium tumefaciens* strain AGL-1, and the transformations for deletion, complementation, or overexpression were performed by ATMT in WT strain or the single-deletion mutants [79], and confirmed by multiple diagnostic PCR. The positive transformants were identified by resistance evaluation and the antibiotic-resistant transformants were purified and finally verified by diagnostic PCR with several specific primer pairs. Primer pairs used in this study are listed in Additional file 2: Table S1.

### Subcellular localization

To generate the GFP-fused strains, the CDS regions of *VdZFP1* and *VdZFP2* without stop codons and GFP fragment were amplified and sequenced, which were inserted into a *Xba*I/*Sac*I-digested pCOM-T0161 vector by homologous recombination of multiple fragments (ClonExpress Ultra One Step Cloning Kit, Vazyme, Nanjing, China). The recombinant plasmids were grown on resistant plates for screening and examined by colony PCR to obtain the recombinant plasmids. The specific primer pairs for amplification and detection purposes related to the above experiments are listed in Additional file 2: Table S1.

Following a published protocol [81], the GFP-labeled strains were obtained by transferring recombinant plasmids into protoplasts of the WT strain. Briefly, the conidia of the WT strain were shaken in CM medium at low speed for 3 days. The hyphae were collected, rinsed, and digested with enzymatic digestion. After 4 h, the digested products were washed and resuspended with NaCl buffer. The protoplasts were adjusted to 5 × 10^6^ mL^−1^ with 1 × STC buffer. Equal volumes of 2 × STC buffer and GFP-fused recombinant plasmids were transferred into 200 μL protoplasts and left at room temperature for 10 min. After 1 mL 50% polyethylene glycol solution was mixed and incubated for 20 min, and 3 mLTB3 liquid media were added for recovering the cell wall of protoplasts. After 8 h, the reconstituted protoplasts were decanted in solid TB3 medium supplemented with G418 to make plates. Before single-spore isolation, resistant transformants were detected by PCR. The transformants were cultured on PDA medium (or supplemented with 0.8 M NaCl) and the GFP signals were observed utilizing a Carl Zeiss Imager.M2 light microscopy system (Jena, Germany).

### Evaluation of morphology, penetration, and microsclerotia formation

All strains used in this study were cultured on PDA at 25 °C in the dark for 5 days. Each experiment was repeated three times independently. The hyphal blocks (1 × 1 mm) cut from the edge of each colony were inoculated on corresponding plates for observation of colony phenotypes, penetration, microsclerotia development, and stress tolerance. All the reagents used were purchased from Solarbio (Beijing Solarbio Science and Technology).

To investigate the melanin phenotypes of the colonies, each strain was cultured on PDA and V8 medium. To identify the stress tolerance of the *VdZFP1* and *VdZFP2* mutant strains, or the double mutant strain, the strains were inoculated on PDA plates with different abiotic factors. Specifically, 4 mM H_2_O_2_ was designed for detecting the sensitivity of WT and mutant strains to oxidative stress. Sorbitol (1 M), 150 μg/mL Congo red, and 0.015% SDS were added for osmotic stress and cell wall integrity assessment. In addition, the colony cross diameters were measured to characterize differences in vegetative growth by inoculating hyphal blocks on Czapek salt medium with different carbon sources, as well as on PDA and V8 medium. All these treatments were cultured in the dark at 25 °C for 7 days. To examine the utilization scytalone, mutant strains were cultured on MM medium supplemented with 50 μg/mL scytalone in the dark at 25 °C for 5 days. Three plates of each medium were inoculated per replicate experiment.

To perform the simulated penetration experiments, hyphal blocks of each strain were incubated on MM medium covered with cellophane membranes for 72 h. The membranes were removed, and the plates were incubated at 25 °C in the dark for another 5 days. Each strain was inoculated on three plates.

The evaluation of microsclerotia development and the extraction of scytalone was referred to in the previous report [39]. Sixty-microliter conidial suspensions (1 × 10^6^/mL) of each strain were coated on BMM plates covered by cellophane membranes (*Ø* = 70 mm) and incubated at 25 °C in the dark. Similarly, melanin biosynthesis inhibitors (60 μg/mL tricyclazole) and the melanin pathway intermediate (50 μg/mL scytalone) were also added to the BMM medium to evaluate the development of microsclerotia. The morphology of microsclerotia was observed under a stereomicroscope at 7 and 14 dpi. Each strain was inoculated on three plates.

To observe the appressoria morphology of *C. gloeosporioides*, conidial suspensions of each strain were adjusted to 100 spores per 10 μL. The conidial suspension (10 μL) of each strain was incubated on hydrophobic glass slides at 25 °C for 20 h in a dark and moisturizing condition. The germination and melanization rate of 120 appressoria were tested.

### Interaction assay

In the yeast one-hybrid (Y1H) system, the full-length CDS regions of *VdCmr1*, *VdZFP1* and *VdZFP2* were amplified and linked into *EcoR*I and *Xho*I-digested pJG4-5 (-prey) vector. Similarly, the upstream promoter fragments (2000 bp) of *VdCmr1* and *VdPKS1* were amplified and inserted into the *Kpn*I and *Xho*I-digested pLacZi2μ (-bait) vector (primer pairs were listed in Additional file 2: Table S1). The bait and prey plasmids were co-transformed into the yeast EYG48 strain and were incubated on SD base medium (without Trp and Ura, Coolaber, Beijing, China) supplemented with 80 μg/mL X-Gal (Coolaber, Beijing, China).

The yeast two-hybrid (Y2H) vectors were prepared by insertion of the respective cDNAs for testing into pGADT7 and pGBKT7. To generate prey and bait constructs, the full-length CDS of *VdCmr1*, *VdZFP1*, and *VdZFP2* were amplified with primer pairs and linked into *EcoR*I-digested vectors, respectively. To test the self-activation function, the recombinant bait plasmids were co-transformed with pGADT7 vector into the yeast Y2Hgold strain and were applied to SD base medium (without Leu and Trp) supplemented with different concentrations of inhibitor 3AT (2.5 to 70 mM). The recombinant bait and prey plasmids were co-transformed into yeast and were cultured on SD base medium (both without Leu, Trp, His, and Ade) supplemented with 20 μg/mL X-a-Gal and 0.1 μg/mL AbA (Coolaber, Beijing, China) to observe the interaction results. The yeast cells containing the pGBKT7-53 and pGADT7-T vectors and pGBKT7-Lam and pGADT7-T vectors were regarded as the positive and negative controls, respectively. The activation functions of pGBKT7-VdZFP1 or -VdZFP2 recombinant plasmids were verified in yeast cells (Y2Hgold) cultured on the SD base medium (both without Trp and His). The pGBKT7-VdCmr1 was used as positive control.

The pHZ65 (YFP^N^/*hyg*) and pHZ68 (YFP^C^/*ble*) vectors were used for the bimolecular fluorescence complementation (BiFC) assays that were conducted as described previously [82]. The full-length CDS regions without stop codons of *VdCmr1*, *VdZFP1*, and *VdZFP2* were cloned into the *Xho*I-digested pHZ65 and pHZ68 vectors to construct VdCmr1-YFP^N^, VdCmr1-YFP^C^, VdZFP1-YFP^N^, VdZFP1-YFP^C^, VdZFP2-YFP^N^, and VdZFP2-YFP^C^ recombinant plasmids by yeast in vivo homologous recombination as previously described [81]. The recombinant plasmids were co-transformed into protoplasts of the WT strain AT13, while each recombinant plasmid co-transformed with pHZ65 or pHZ68 vectors were treated as controls. The transformants were screened on PDA medium supplemented with 50 μg/mL hygromycin and 250 μg/mL zeocin. Before single-spore isolation, all the resistant transformants were confirmed by PCR. The transformants were treated with the cell wall dye CFW (calcofluorwhite, 0.5 g/L; Sigma), and the fluorescent signals were observed utilizing a Carl Zeiss Imager.M2 light microscopy system. Each interaction analysis was repeated 3 times.

### Pathogenicity assay

Pathogenicity assays were performed using a root-dip inoculation method [47]. Before inoculation, susceptible cotton (Junmian No.1), tobacco, and Shantung maple seedlings were grown in a greenhouse at 25 °C for about 3, 4, and 12 weeks, respectively. The conidial suspensions of each strain were obtained by filtration, centrifugation, washing, and dilution after shaking in liquid CM medium. Seedlings were removed from the soil medium and washed. Their roots were immersed in the conidial suspensions for 30 min. The cotton and tobacco roots were immersed in a concentration of 5 × 10^6^ spores/mL while the maple roots were immersed in a dilution of 5 × 10^7^ spores/mL. Each strain was inoculated in three replicated experiments with 20 cotton, 6 tobacco, or 10 maple seedlings in each experiment, while the treatments of water and WT strain were used as negative and positive controls, respectively.

Considering the different disease cycles of each host, we observed the disease phenotypes and collected samples for fungal biomass analyses on the 21st, 18th, and 50th days after inoculation of cotton, tobacco, and maple trees, respectively. Subsequently, to characterize the pathogenicity of each strain on maple seedlings, disease severity scores were divided into four categories: 0 = healthy; 1 = one true leaf showing yellowing; 2 = two or three true leaves showing wilt symptoms; 3 = all leaves wilted and even plant dead (disease index was calculated as [70] described).

To examine pathogenicity in *C. gloeosporioides*, conidial suspensions (1 × 10^6^ spores/mL) of each strain were obtained by shaking in PDB medium at 25 °C for 2 days. The leaves of *Liriodendron chinense* collected in Nanjing Forestry University were washed with tap water, and conidial suspensions were inoculated on leaves after being punctured at 25 °C in the dark for 4 days. This experiment was repeated three times, with 6 leaves inoculated in each experiment.

### Analysis of relative gene expression and fungal biomass

To detect the fungal biomass in the host, the stems of plants were collected after pathogenicity assays. To examine the expression profile of *VdZFP1* and *VdZFP2* during microsclerotia formation, 20 μL of a conidial suspension of 5 × 10^6^ spores /mL of WT strain were incubated on the cellophane cellophanes covered on the BMM plates and the samples were collected at 2, 3, 4, 5, 7, and 14 dpi described in previous study [57]. The different gene expression levels among mutants, complemented strains, or overexpressing strains were detected by comparing the expression levels of target genes in the WT strain and mutants cultured on BMM medium for 5 days in the dark. Similarly, samples were collected on BMM medium with 50 μg/mL scytalone for detecting the expression of melanin-related genes.

All plant/fungal samples were prepared in advance and stored at − 80 °C until use. The genes in the melanin biosynthesis pathway were those characterized by [9], including *T4HR*, *SCD*, *T3HR*, *VdPKS1*, *VdCmr1*, and *VdLac1*. Total RNA or DNA for the expression or biomass analyses were extracted by reagent kit (TIANGEN, Beijing, China). Reverse transcription-quantitative PCR (RT-qPCR) and qPCR were performed to analyze gene expression and fungal biomass, respectively. In the fungal biomass analysis, qPCR was carried out with the cotton and maple 18S rDNA gene (*Gh18S* and *At18S*) and tobacco *NbEF* as internal plant reference genes to quantify DNA of *V. dahliae* using the target of elongation factor 1α gene *VdEF-1α*. For analyses of relative expression, the *VdEF-1α* was used for normalization. The primer pairs of these experiments are listed in Additional file 2: Table S1.

The amplification reactions were carried out using 2 × Top Green qPCR SuperMix (TransGen Biotech, Beijing, China) and the QuantStudio3 Real-Time PCR system (Thermo Fisher Scientific, USA). The reaction volumes of both RT-qPCR and qPCR were 20 μL (10 μL SuperMix, 1.2 μL primer pair, 1.5 μL cDNA or gDNA, and 7.3 μL ddH_2_O) and their cycling procedures included pre-denaturation at 95 °C for 3 min, followed by 40 cycles of 95 °C denaturation for 15 s, 60 °C annealing for 20 s, and 72 °C extension for 20 s. There were three replicates for the qPCR and RT-qPCR experiments, enabling calculations of mean and standard error. The 2^−ΔΔCT^ method [83] was used to calculate the relative expression levels or pathogen biomass contents.

### Statistical analysis

The mean ± SD was calculated for each treatment with three or more replicates. Significant differences of the growth and inhibition rate on corresponding plates, as well as the numbers, volume, and melanin coverage of microsclerotia were analyzed by ordinary Student’s *t* test in the Microsoft Excel software. Additionally, significant differences of gene expression levels and fungal biomasses among treatments were identified using one-way analysis of variance (ANOVA) followed by least significant difference mean separation tests. Statistical analyses were performed using the SPSS version 19 software package (SPSS Inc., Chicago, IL, USA). To visually display the differences, the expression levels of *VdZFP1* and *VdZFP2* during microsclerotia formation were converted to heat maps using HemI (Heatmap Illustrator, version 1.0).

### Supplementary Information

**Additional file 1: Figure S1.** Verification of *VdZFP1* and/or *VdZFP2* mutants and complemented strains. **Figure S2.** The regulatory relationship between *VdZFPs*, and their response to microsclerotia induction of *V. dahliae* Vd991 strain. **Figure S3.** VdZFP1 involves in vegetative growth of *V. dahliae*. **Figure S4.** Overexpression of *VdPKS9* in *VdZFP1* or *VdZFP2* mutant background reduces melanin biosynthesis. **Figure S5.** The supplement of scytalone recovers melanin biosynthesis of albino strains. **Figure S6.** *V. dahliae VdZFP1* and *VdZFP2* positively regulate *VdCmr1*. **Figure S7.** *V. dahliae VdZFP1* and *VdZFP2* are dispensable for pathogenicity. **Figure S8.** A homologous fragment containing *VdZFPs* and adjacent genes are highly similar between *V. dahliae* and *C. gloeosporioides*. **Figure S9.** The functions analysis of VdZFP1 and VdZFP2 homologs of *C. gloeosporioides*. **Figure S10.** Upstream starvation signaling and Hog-MAPK pathway elements regulate VdZFP1 and VdZFP2 in *V. dahliae*.

**Additional file 2: Table S1.** Information on the primer pairs used to construct the vector in this study. **Table S2.** Information of RT-qPCR primer pairs used in this study.

**Additional file 3. **Raw data of experimental results.

## Data Availability

All study data are included in the article and/or supplemental information.

## Abbreviations

Y1H/Y2H: Yeast one-/two-hybrid
BiFC: Bimolecular fluorescence complementation
DHN: 1,8-Dihydroxynaphthalene
L-DOPA: L-3,4-dihydroxyphenylalanine
PKS: Polyketide synthases
1,3,6,8-THN: 1,3,6,8-Tetrahydroxynaphthalene
1,3,8-THN: 1,3,8-Trihydroxynaphthalene
TFs: Transcription factors
MAPK: Mitogen-activated protein kinase
PKA: Protein kinase A
ROS/RNS: Reactive oxygen/nitrogen species
ZFP: Zinc finger proteins
GFP/YFP: Green/yellow fluorescent protein
WT: Wild type
EC: Ectopic complemented
dpi/hpi: Days/hours post inoculation
OE: Overexpression
3AT: 3-Amino-1,2,4-triazole
AbA: Aureobasidin A
X-α-Gal: 5-Bromo-4-chloro-3-indolyl-α-D-galactopyranoside
H_2_O_2_: Hydrogen peroxide
SDS: Sodium dodecyl sulfate
PDA: Potato dextrose agar
CM: Complete medium
ATMT: *Agrobactirium tumfacience* Mediated-transformant
CFW: Calcofluorwhite
