# Supplementary material for: Two zinc finger proteins, VdZFP1 and VdZFP2, interact with VdCmr1 to promote melanized microsclerotia development and stress tolerance in Verticillium dahliae
Source: BMC Biol. 2023 Oct 31;21:237. doi: 10.1186/s12915-023-01697-w (PMC10617112; doi:10.1186/s12915-023-01697-w)
Supplement: Supplementary file 1 — Additional file 1: Figure S1. Verification of VdZFP1 and/or VdZFP2 mutants and complemented strains. Figure S2. The regulatory relationship between VdZFPs, and their response to microsclerotia induction of V. dahliae Vd991 strain. Figure S3. VdZFP1 involves in vegetative growth of V. dahliae. Figure S4. Overexpression of VdPKS9 in VdZFP1 or VdZFP2 mutant background reduces melanin biosynthesis. Figure S5. The supplement of scytalone recovers melanin biosynthesis of albino strains. Figure S6. V. dahliae VdZFP1 and VdZFP2 positively regulate VdCmr1. Figure S7. V. dahliae VdZFP1 and VdZFP2 are dispensable for pathogenicity. Figure S8. A homologous fragment containing VdZFPs and adjacent genes are highly similar between V. dahliae and C. gloeosporioides. Figure S9. The functions analysis of VdZFP1 and VdZFP2 homologs of C. gloeosporioides. Figure S10. Upstream starvation signaling and Hog-MAPK pathway elements regulate VdZFP1 and VdZFP2 in V. dahliae. [file 12915_2023_1697_MOESM1_ESM.docx]

# Additional file 1: Supplementary Figures

## Figure S1


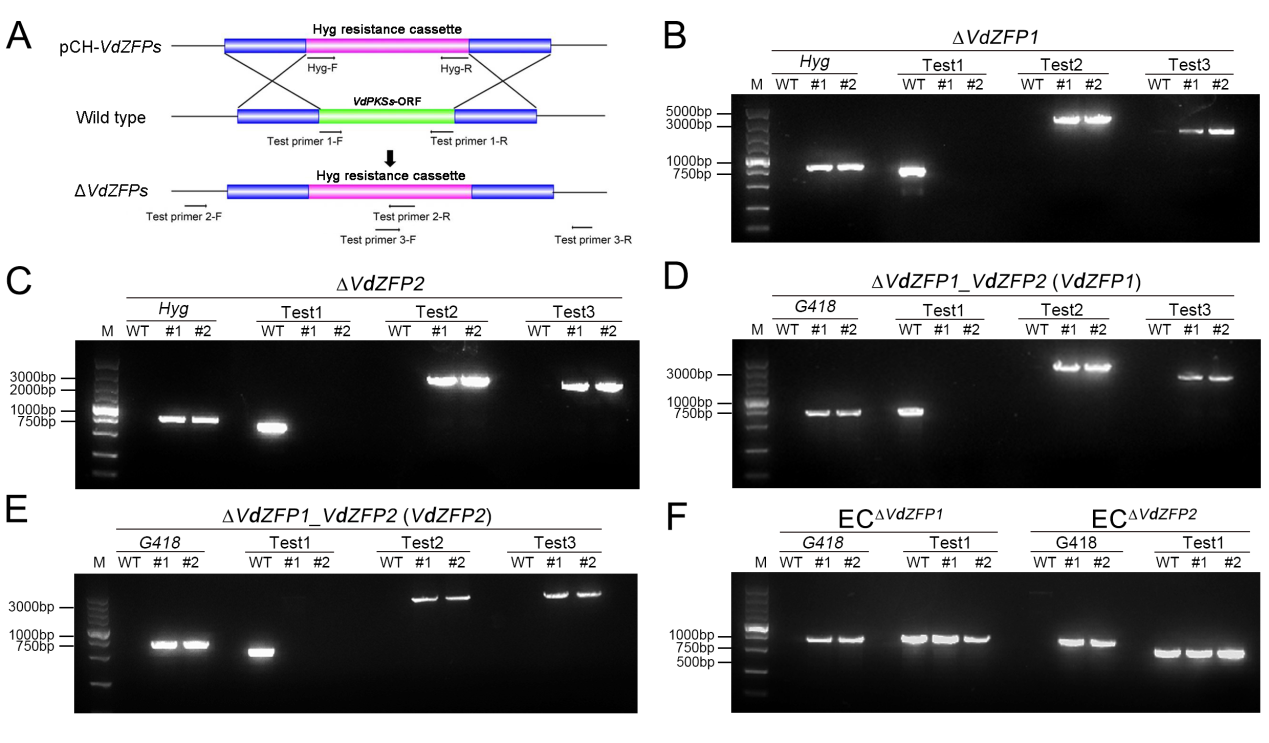


**Figure S1 | Verification of *VdZFP1* and/or *VdZFP2* mutants and complemented strains.**

**(A)** Schematic diagram of *VdZFP1* and/or *VdZFP2* knockout strategy. The *VdZFP1* and/or *VdZFP2* were deleted by homologous recombination. In single-deletion mutant, target gene was replaced by *Hyg* resistance cassette, while *G418* resistance cassette was introduced in *VdZFP1* or *VdZFP2* mutant to replace another gene.

**(B-F)** Diagnostic PCR of *VdZFP1* and/or *VdZFP2* mutants and complemented strains. All strains were detected with multiple specific primer pairs shown in (A), and the amplified fragments were indicated by agarose gel electrophoresis with WT strain as control. **(B)** and **(C)** detection of *VdZFP1* or *VdZFP2* mutants, **(D)** and **(E)** detection of double-deleted mutants, **(F)** detection of complemented strains.

## Figure S2


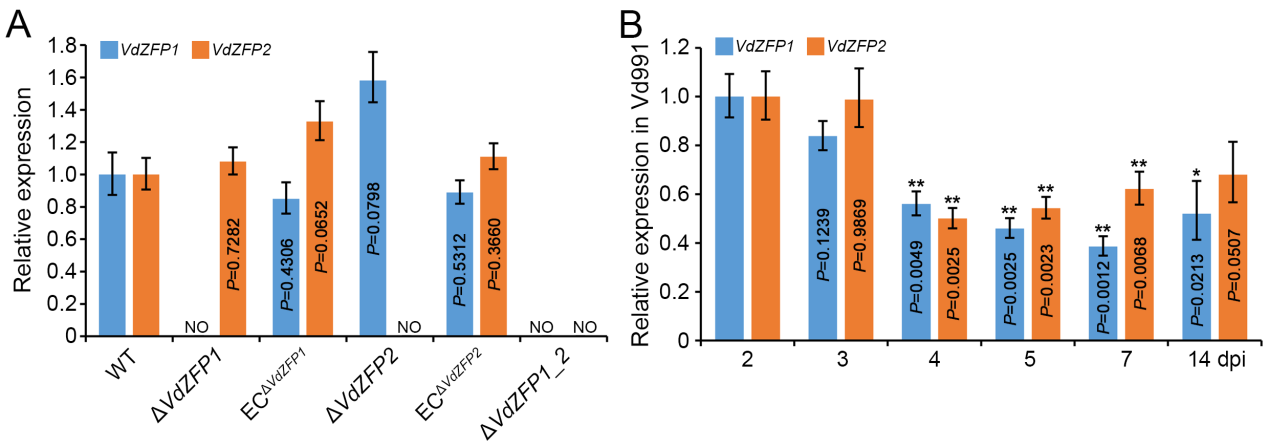


**Figure S2 | The regulatory relationship between *VdZFPs*, and their response to microsclerotia induction of *V. dahliae* Vd991 strain.**

**(A)** Relative expression of *VdZFP1* and *VdZFP2* in WT, Δ*VdZFP1*, EC^Δ^*^VdZFP1^*, Δ*VdZFP2* and EC^Δ^*^VdZFP2^* strains. The RNA samples of indicated strains were collected on PDA medium 5 days after incubating at 25°C in the dark.

**(B)** Expression profile of *VdZFP1* and *VdZFP2* during microsclerotia development of *V. dahliae* Vd991 strain. The WT strain were cultured on BMM medium covered with cellophane membranes at 25 °C in the dark and samples were collected at 2, 3, 4, 5, 7 and 14 dpi. In these experiments, the relative expression of *VdZFP1* and *VdZFP2* were calculated from the RT-qPCR results using the 2^-ΔΔCT^ method with WT strain or 2 dpi sample as control. These experiments were independently repeated 3 times to determine the trend.

## Figure S3


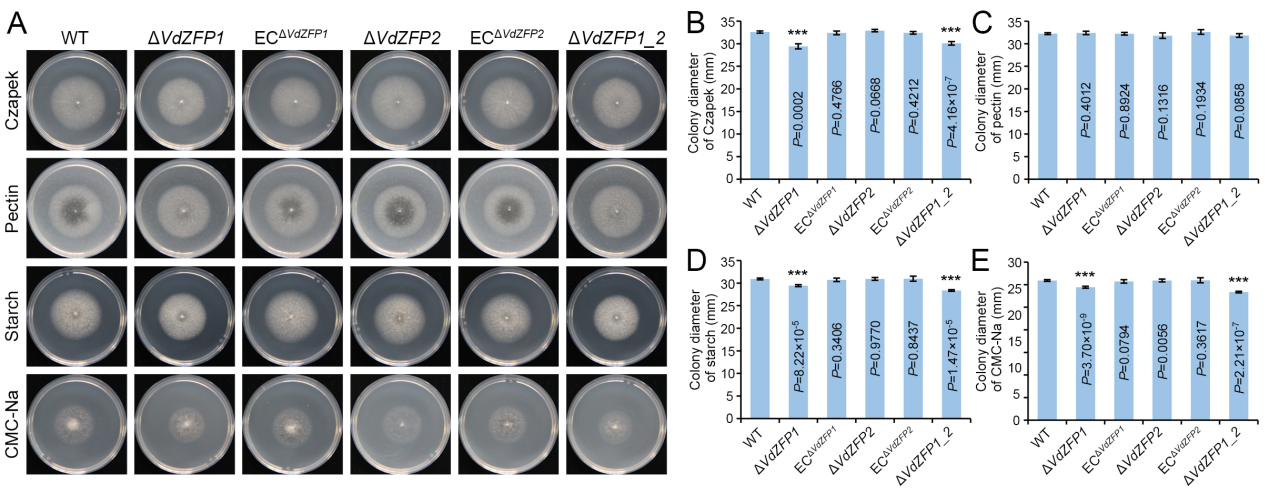


**Figure S3 | VdZFP1 involves in vegetative growth of *V. dahliae*.**

**(A)** Colony morphology of WT, Δ*VdZFP1*, EC^Δ^*^VdZFP1^*, Δ*VdZFP2*, EC^Δ^*^VdZFP1^* and Δ*VdZFP1_2* strains growth on medium containing various carbon source. These strains grown on Czapek salt medium that supplemented with sucrose, pectin, starch and sodium carboxymethyl cellulose as carbon source. The phenotypes were photographed 7 days after incubation at 25°C in the dark. Each strain was inoculated at least 3 plates and repeated 3 times independently.

**(B-E)** Colony diameter of the indicated strains in panel (A). Error bars are standard errors calculated from six replicates, and this experiment performed three repeats. ^***^*P* < 0.001 (Student’s *t*-test).

## Figure S4


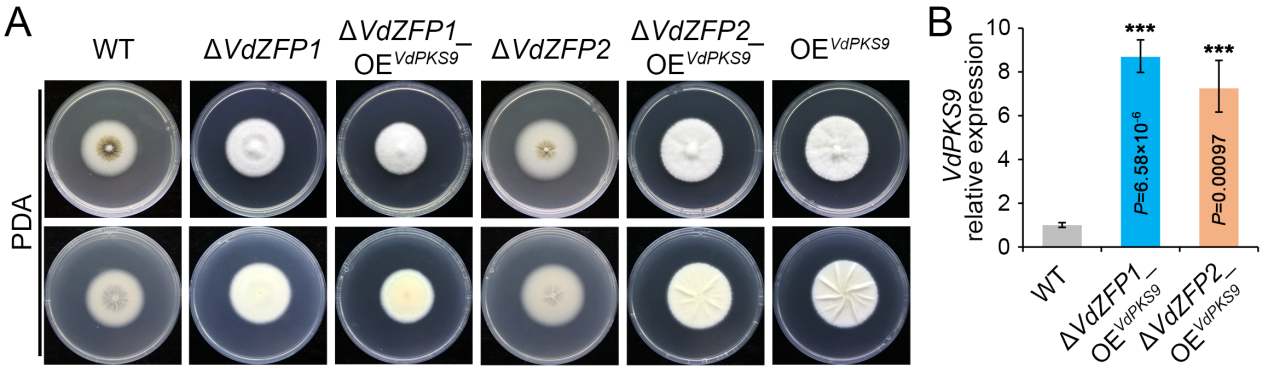


**Figure S4 | Overexpression of *VdPKS9* in *VdZFP1* or *VdZFP2* mutant background reduces melanin biosynthesis.**

**(A)** Colony phenotypes of WT, Δ*VdZFP1*, Δ*VdZFP1_*OE*^VdPKS9^*, Δ*VdZFP2*, Δ*VdZFP2_*OE*^VdPKS9^* and OE*^VdPKS9^* strains inoculated on PDA medium at 25 °C in the dark. The phenotypes were photographed 7 days after incubation. Each strain was inoculated at least three plates and three independent experiments were carried out.

(**B)** Detection of *VdPKS9* overexpression level in indicated strains. The samples of WT, Δ*VdZFP1_*OE*^VdPKS9^* and Δ*VdZFP2_*OE*^VdPKS9^* strains were collected on BMM medium 5 days after incubating at 25°C in the dark. The relative expression of *VdPKS9* was calculated from the RT-qPCR results using the 2^-ΔΔCT^ method with WT strain as control and independently repeated 3 times.

## Figure S5


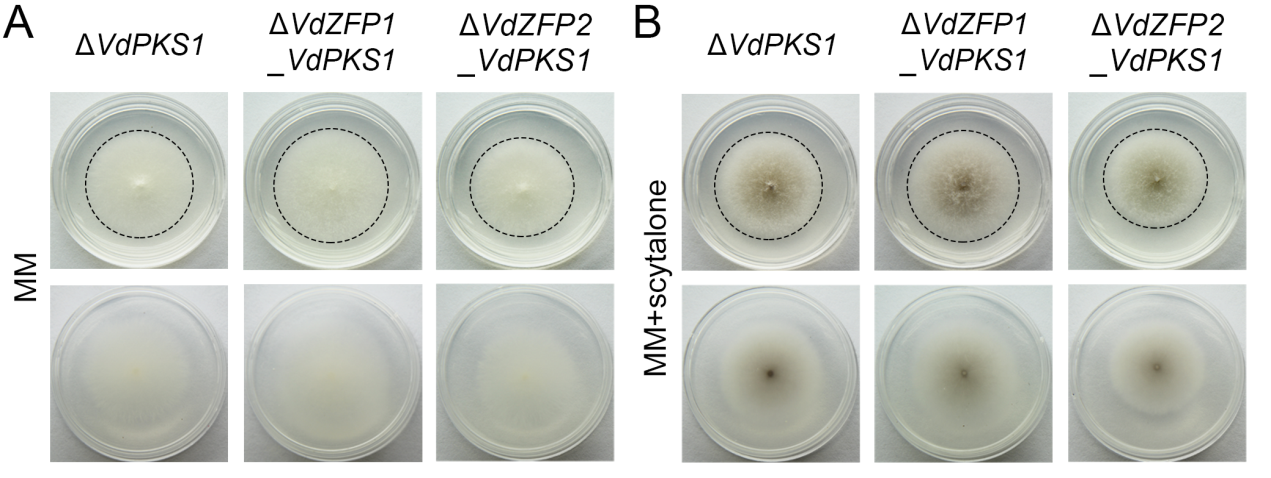


**Figure S5 | The supplement of scytalone recovers melanin biosynthesis of albino strains.**

**(A) and (B)** Investigation of melanin biosynthesis in Δ*VdPKS1* and double-deleted mutants (Δ*VdZFP1_VdPKS1* and Δ*VdZFP2_VdPKS1*). The phenotypes of indicated strains were photographed 5 days after incubation on MM medium **(A)** or supplemented with 50 mg/mL scytalone **(B)** at 25 °C in the dark. Each strain was inoculated at least three plates and three independent experiments were carried out.

## Figure S6


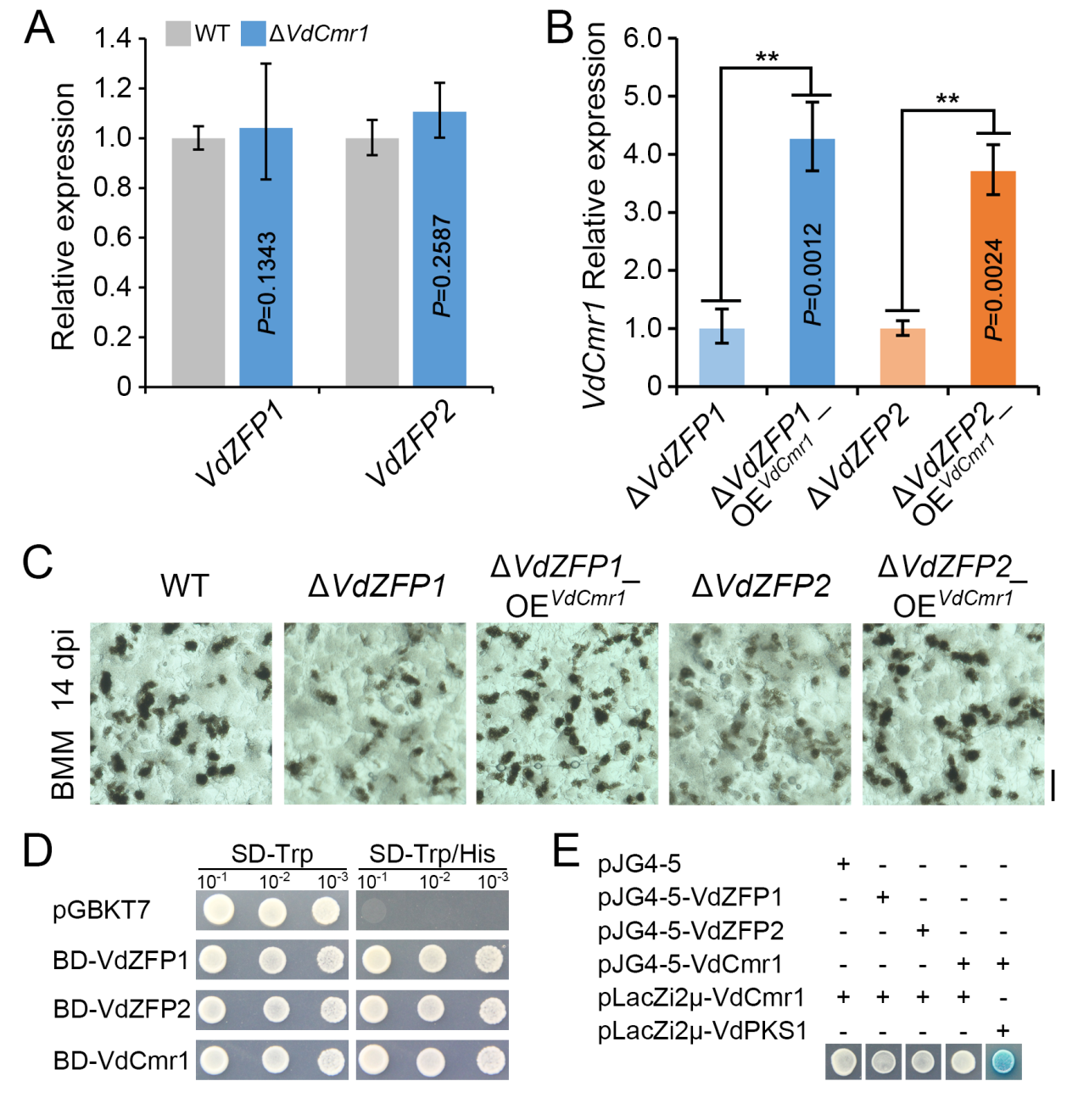


**Figure S6 | *V. dahliae VdZFP1* and *VdZFP2* positively regulate *VdCmr1*.**

**(A)** Relative expression of *VdZFP1* and *VdZFP2* in Δ*VdCmr1* mutant. The samples of WT and Δ*VdCmr1* strains were collected on BMM medium 5 days after incubating at 25°C in the dark.

(**B)** Detection of *VdCmr1* overexpression level in indicated strains. The samples of Δ*VdZFP1*, Δ*VdZFP1_*OE*^VdCmr1^*, Δ*VdZFP2* and Δ*VdZFP2_*OE*^VdCmr1^* strains were collected on BMM medium 5 days after incubating at 25°C in the dark. The relative expression of *VdZFPs* and *VdPKS9* were calculated from the RT-qPCR results using the 2^-ΔΔCT^ method with WT strain or Δ*VdZFP1*(or Δ*VdZFP2*) mutant as control and independently repeated 3 times.

**(C)** Microsclerotia morphology of indicated strains. The microsclerotia development of each strain was observed at 14 dpi after incubating on the BMM plates covered with cellophane membranes at 25°C in the dark and photographed with a stereoscope. Each strain was repeated three times independently, and at least three plates were observed each time. Scale bar =100 μm.

**(D)** Activation analyses among VdZFP1, VdZFP2 and VdCmr1 in a yeast two-hybrid system. Their CDS regions were inserted into pGBKT7 vectors to obtain the bait constructs. Each bait construct was transformed into yeast cells. Yeast cells with 10-fold serial dilutions were cultured on SD base medium (lacking Trp or Trp/His). The yeast cells transformed with pGBKT7 were the negative control. The Interaction phenotypes were photographed at 3 dpi. This experiment was repeated 3 times.

**(E)** Promoter-binding assays among *VdZFP1*, *VdZFP2* and *VdCmr1* in a yeast one-hybrid (Y1H) system. The full-length CDS regions of three genes were inserted into a pJG4-5 vector, while the promoter fragments of *VdCmr1* and *VdPKS1* were inserted into a pLacZi2μ vector. The bait and prey plasmids were co-transformed into the yeast EYG48 strain and were incubated on SD base medium (without Trp and Ura) supplemented with 80 μg/mL X-Gal. The yeast cells co-transformed with pJG4-5-VdCmr1 and pLacZi2μ-VdPKS1 were the positive control. This experiment was repeated 3 times.

## Figure S7


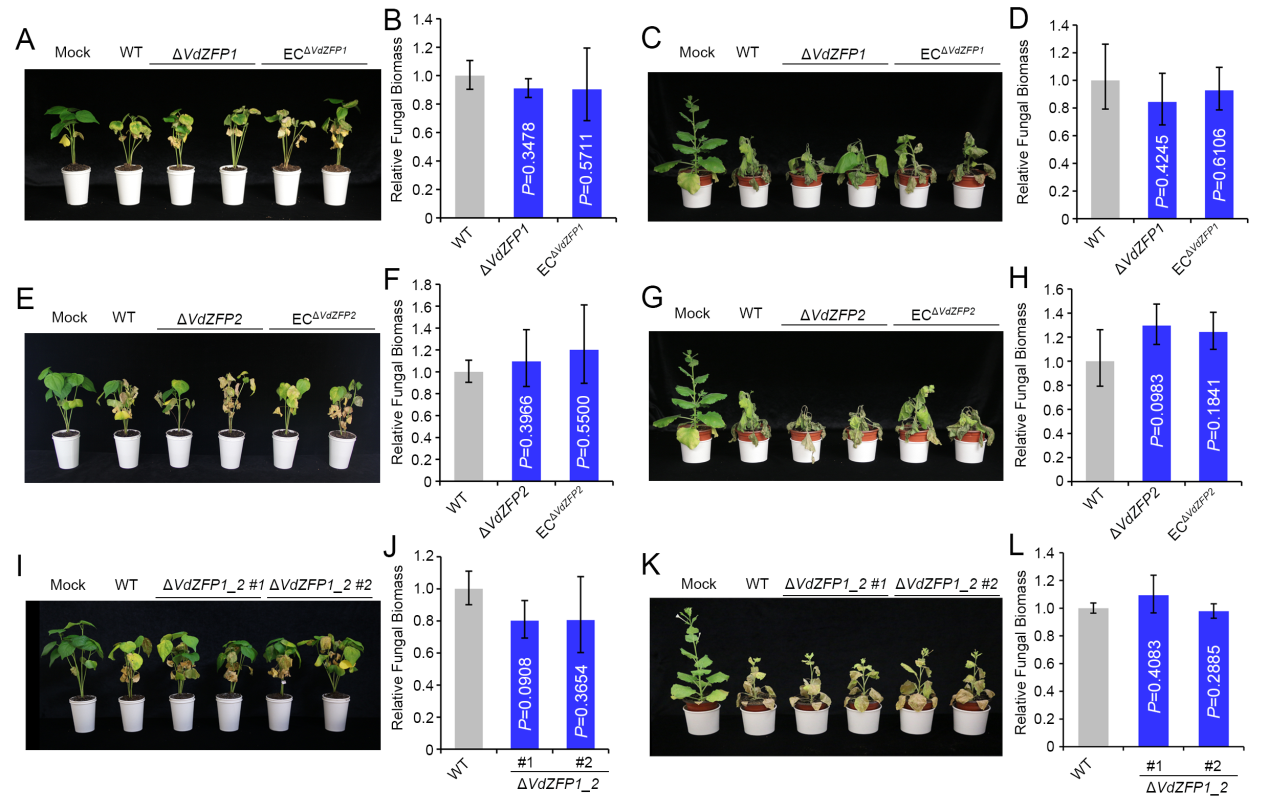


**Figure S7 | *V. dahliae* VdZFP1 and VdZFP2 are dispensable for pathogenicity.**

**(A), (C), (E), (G), (I) and (K)** Pathogenicity assay of the indicated strains on cotton and tobacco. Seedlings were inoculated with WT, Δ*VdZFP1*, EC^Δ^*^VdZFP1^*, Δ*VdZFP2*, EC^Δ^*^VdZFP1^* and Δ*VdZFP1_2* strains, while the H_2_O treatment was set as negative control. The Verticillium wilt symptom were photographed at 21 and 18 dpi.

**(B), (D), (F), (H), (J) and (L)** Quantification the fungal biomass in cotton and tobacco stems by qPCR following inoculation of the indicated strains. Samples were collected from the stem base of infected plants at 21 and 18 dpi. The *Gh18S* of cotton and *NbEF* of tobacco were served as an endogenous control to evaluate the endophytic colonization of *V. dahliae* by quantifying *VdEF-1α*. The pathogenicity was analyzed with three replicates (60 cotton and 18 tobacco seedlings), and the fungal biomass was calculated by three independent biological replicates. Error bars represent standard errors. *P* > 0.05 means no significantly (one-way ANOVA).

## Figure S8


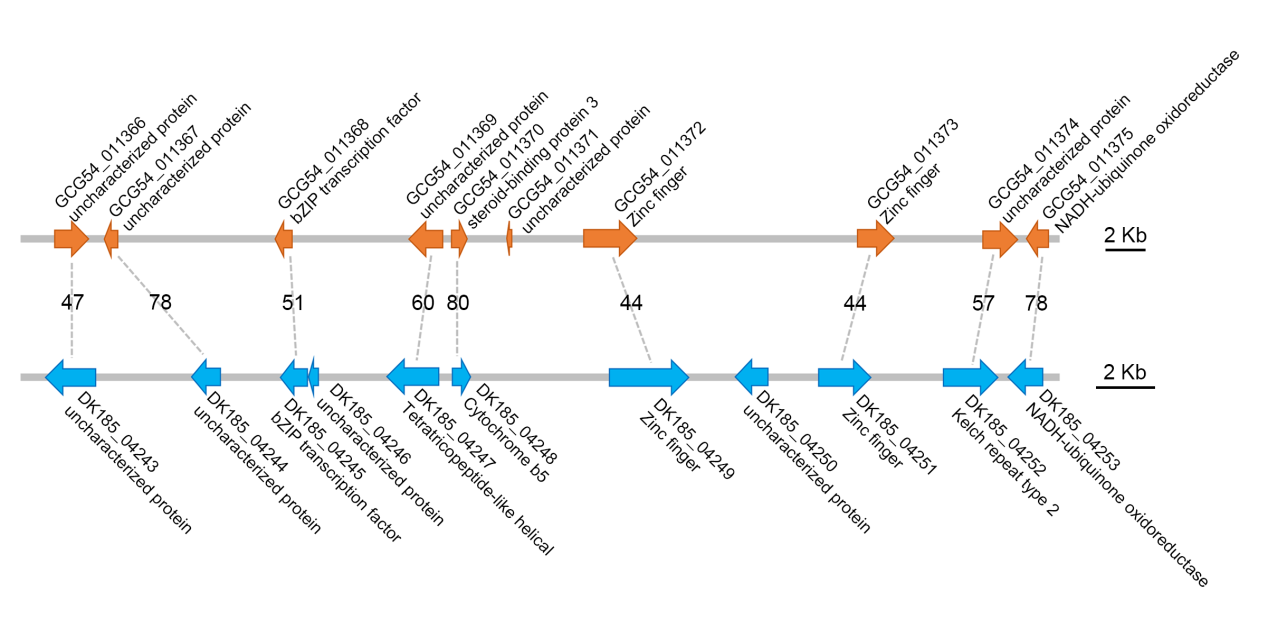


**Figure S8 | A homologous fragment containing *VdZFPs*** **and adjacent genes are highly similar between** ***V. dahliae* and *C. gloeosporioides*.**

Gene collinearity and functional analysis of fragments containing VdZFP1 and VdZFP2 homologues in *V. dahliae* and *C. gloeosporioides* were determined on NCBI database. The arrows represent the direction of expression, while the homologies were noted on dashed lines.

## Figure S9


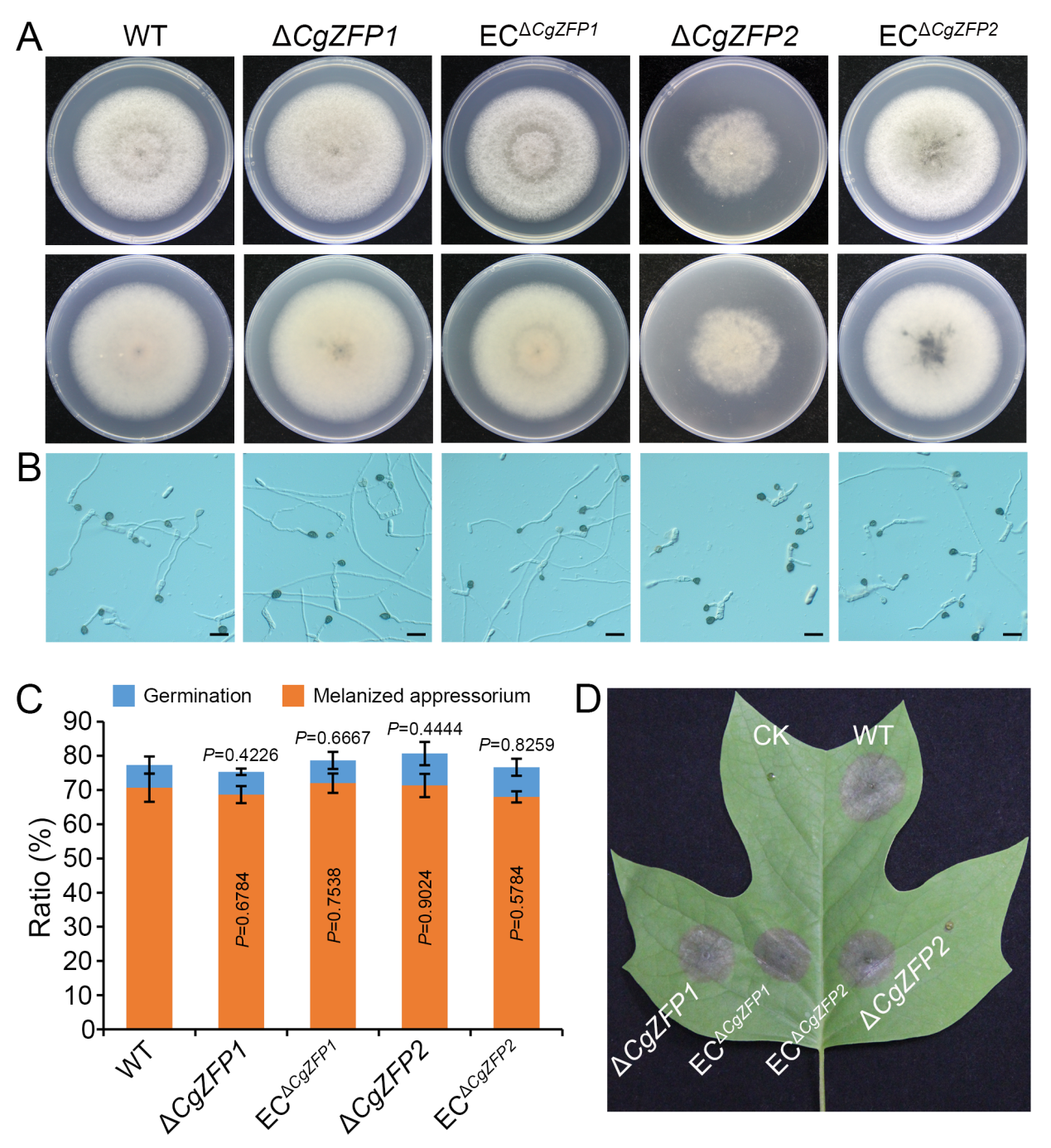


**Figure S9 |** **The functions analysis of VdZFP1 and VdZFP2 homologs of *C. gloeosporioides*.**

**(A)** Colony morphology of *C. gloeosporioides* WT, Δ*CgZFP1*, EC^Δ^*^CgZFP1^*, Δ*CgZFP2*, and EC^Δ^*^CgZFP1^* strains growth on PDA medium. The phenotypes were photographed 7 days after incubation at 25°C in the dark. Each strain was inoculated at least 3 plates and repeated 3 times independently.

**(B)** Melanized appressoria of indicated strains. The WT, Δ*CgZFP1*, EC^Δ^*^CgZFP1^*, Δ*CgZFP2*, and EC^Δ^*^CgZFP1^* strains were incubated on hydrophobic slides at 25°C in the dark for 16 hours. The morphology of appressoria were observed by a light microscope. Scale bar =20 μm.

**(C)** Conidial germination rate and proportion of melanized appressoria of WT, Δ*CgZFP1*, EC^Δ^*^CgZFP1^*, Δ*CgZFP2*, and EC^Δ^*^CgZFP1^* strains. The results were calculated from panel (B). Error bars are standard errors calculated from six replicates, and this experiment performed three repeats, *P* > 0.05 means no significantly different (Student’s *t*-test).

(D) Pathogenicity assay of the indicated strains on *Liriodendron chinense*. The conidial suspension of WT, Δ*CgZFP1*, EC^Δ^*^CgZFP1^*, Δ*CgZFP2*, and EC^Δ^*^CgZFP1^* strains were inoculated on leaves after being punctured at 25 °C in the dark for 4 days. This experiment was repeated 3 times, with 6 leaves inoculated each time.

## Figure S10


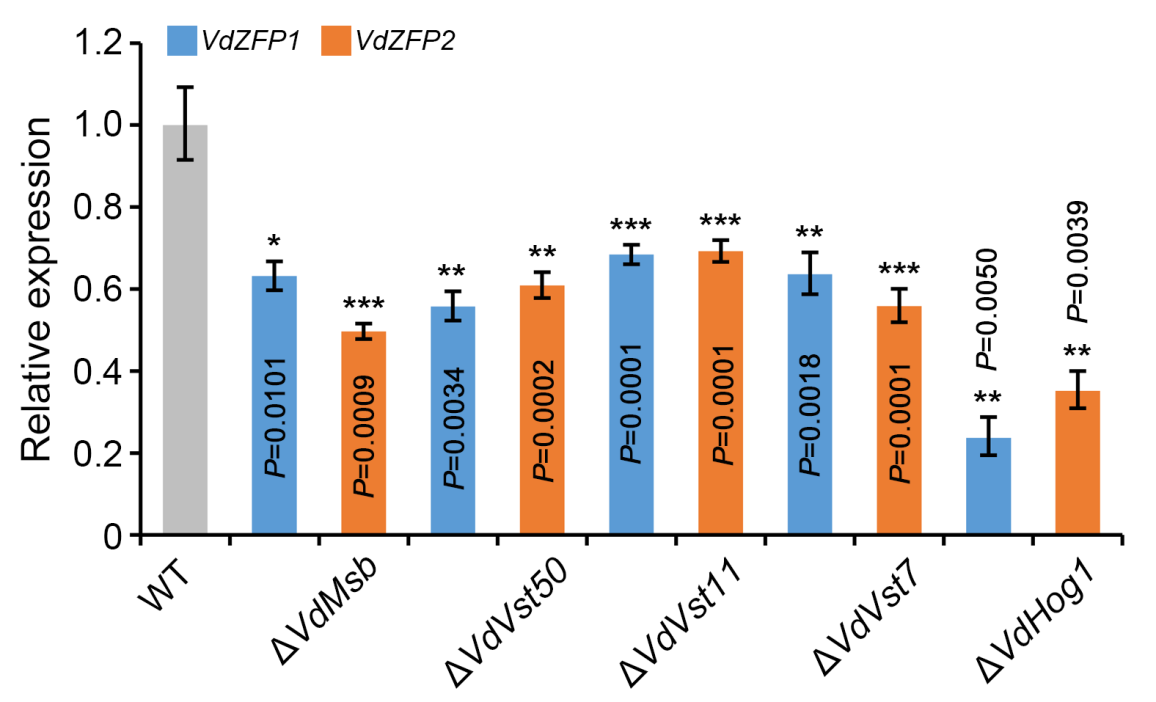


**Figure S10 |** **Upstream starvation signaling and Hog-MAPK pathway elements regulate VdZFP1 and VdZFP2 in *V. dahliae*.**

Relative expression of *VdZFP1* and *VdZFP2* in indicated strains. These strains (WT, Δ*VdMsb*, Δ*VdVst50*, Δ*VdVst11*, Δ*VdVst7* and Δ*VdHog1*) were collected after induction on BMM medium at 25 °C in the dark for 5 days. The results were detected by RT-qPCR and conducted using the 2^-ΔΔCT^ method. This experiment independently repeated 3 times. Error bars represent standard errors of the mean, ^*^*P* < 0.05, ^**^*P* < 0.01, and ^***^*P* < 0.001 (one-way ANOVA).
